# Clinical prediction models for febrile neutropenia and its outcomes: a systematic review

**DOI:** 10.1007/s00520-025-09562-y

**Published:** 2025-06-04

**Authors:** Joshua Sheehy, Marianne Gallanagh, Clair Sullivan, Steven Lane

**Affiliations:** 1 Haematology and Bone Marrow Transplant Department, Cancer Care Services, The Royal Brisbane and Women’s Hospital, Brisbane, QLD Australia; 2 Faculty of Medicine, The University of Queensland, Brisbane, QLD Australia; 3 QIMR Berghofer Medical Research Institute, Brisbane, QLD Australia; 4 Metro North Hospital and Health Service, Department of Health, Queensland Government, Brisbane, QLD Australia; 5 Queensland Digital Health Centre, Faculty of Medicine, The University of Queensland, Brisbane, QLD Australia

**Keywords:** Febrile neutropenia, Prediction model, Risk score

## Abstract

**Purpose:**

Febrile neutropenia (FN) is a life-threatening complication of chemotherapy. Although practice guidelines suggest the use of existing prediction models when making decisions to prevent and treat FN, recent evidence suggests that these models are limited in their discriminative ability. This study aims to systematically review and critically evaluate the recent literature to assess the question: what evidence-based clinical prediction models can be used to predict FN or its outcomes?

**Methods:**

PubMed, EMBASE, Web of Science, and SCOPUS were searched for primary journal articles that developed or validated models that predicted FN or outcomes in patients with FN. Risk of bias was critically evaluated using the Prediction model Risk of Bias Assessment Tool (PROBAST).

**Results:**

Five thousand nine hundred nineteen articles were identified, of which 90 met inclusion criteria. Twenty-five studies predicted FN, and 65 studies predicted outcomes in patients with FN, including 28 that predicted mortality, 35 that predicted microbiological outcomes, and 35 that predicted other complications. Eight studies used machine learning methods in their development, and few studies were externally validated. All 90 studies were graded as high risk of bias using PROBAST.

**Conclusion:**

Prediction models for FN and its outcomes demonstrate promising discriminatory ability; however, several limitations have prevented these from translating clinically. These limitations include variable FN definitions, high ROB in current models, limited external validation, and heterogeneous cohorts. Future work is needed to further develop and validate robust, well-evidenced models that can translate into clinical practice. This may best be achieved through machine learning and electronic medical record integration.

**Supplementary Information:**

The online version contains supplementary material available at 10.1007/s00520-025-09562-y.

## Introduction

Febrile neutropenia (FN) is a common and often life-threatening condition in cancer patients undergoing chemotherapy treatment. FN affects up to 20% of patients and has a mortality rate of 9.5% [1]. This mortality increases to 50% in those with comorbidities or septic shock [2]. FN is defined as fever ≥ 38.3 °C or ≥ 38.0 °C for 1 h or more, and neutropenia is defined as an absolute neutrophil count (ANC) of < 0.5 × 10^9^/L, or an ANC that is < 1.0 × 10^9^/L and expected to decrease to < 0.5 × 10^9^ in the next 48 h [3].

Given the significant complications that can occur with FN, high-risk patients are given granulocyte colony stimulating factor (G-CSF), and in some cases, prophylactic antibiotics. At the onset of FN, standard of care tends to be inpatient management with broad-spectrum intravenous antibiotics, but this treatment paradigm is not without risk, notably the development of nosocomial colonization and resistant bacterial organisms [4]. There is a significant economic impact on the healthcare system with this approach to management, which prompts the question of whether there is a cohort of FN patients that can be safely managed in the outpatient setting [5].

Multiple risk stratification tools have been developed to both determine prognosis and to tailor therapy in patients with FN. The most internationally utilized prediction tools to predict poor outcomes in FN are the Multinational Association for Supportive Care in Cancer (MASCC) score and the Clinical Index of Stable Febrile Neutropenia (CISNE) [6, 7]. Although these scoring systems performed well in initial validation, a recent systematic review and meta-analysis demonstrated that these tools may not meet the appropriate safety standards for clinical decision making [8]. International guidelines recommend incorporating clinical criteria when making decisions, in conjunction with these tools, further implying the need for more comprehensive predictive tools to aid clinical decision making in this setting [5]. With the increasing use of electronic medical records (EMR) globally and subsequent availability of large datasets, there is growing opportunity for the development of novel models. Furthermore, machine learning (ML) algorithms present a rapidly expanding area of development and may represent an opportunity for more discriminate models to be developed with these large datasets.

Given the importance of early intervention in FN, and the lack of validated, fit-for-purpose prediction models clinically available, novel prediction models are needed. Our aim was to systematically review and critically appraise the existing and emerging literature with the following question: what evidence-based clinical prediction models can be used to predict FN or its outcomes?

## Methods

### Search strategy

The Preferred Reporting Items for Systematic Reviews and Meta-Analyses (PRISMA) guidelines were followed. Searches were performed in four databases (PubMed, Embase, Web of Science, and SCOPUS) for studies that developed or validated models predicting febrile neutropenia or its outcomes. The search strategy was developed in PubMed using medical subject headings (MeSH) and keyword terms. It was then revised for the other databases (Supplementary Material). The final iteration of the search was completed on October 5, 2023. A protocol was not pre-registered for this review.

### Eligibility criteria

Articles published between 2013 and 2023 were evaluated. Studies that evaluated the use of a predictive model to predict FN or its outcomes in a cohort that included those age ≥ 18 years were included. The following papers were excluded; studies that did not develop a predictive model, abstracts only, studies not available in English, and conference papers. Title and abstract screening, full-text review, data extraction, and risk of bias (ROB) assessment was performed and corroborated by two independent authors using Covidence, a systematic review software tool.

### Data extraction and ROB assessment

Each study was systematically reviewed for data using a structured proforma. Data were collected pertaining to study characteristics, dataset, cohort demographics including antimicrobial and G-CSF prophylaxis, predictor variables, intended time of use, endpoints, outcome measures, and validation. Predictors were classified as clinical, pathological, radiological, or microbiological, and endpoints were classified as predicting FN, mortality, microbiological outcomes, complications, or other outcomes of interest.

ROB and applicability were assessed using the PROBAST (Prediction model Risk of Bias Assessment Tool), which is a tool developed specifically for assessing prediction models in the systematic review and meta-analysis setting. The tool assesses ROB across four domains (participants, predictors, outcomes, and analysis) and for the study overall. Two independent authors assessed ROB using the PROBAST. Where disagreements were identified, these were resolved first through discussion between the authors. There were no cases where discussion did not resolve these conflicts.

## Results

**Fig. 1:**
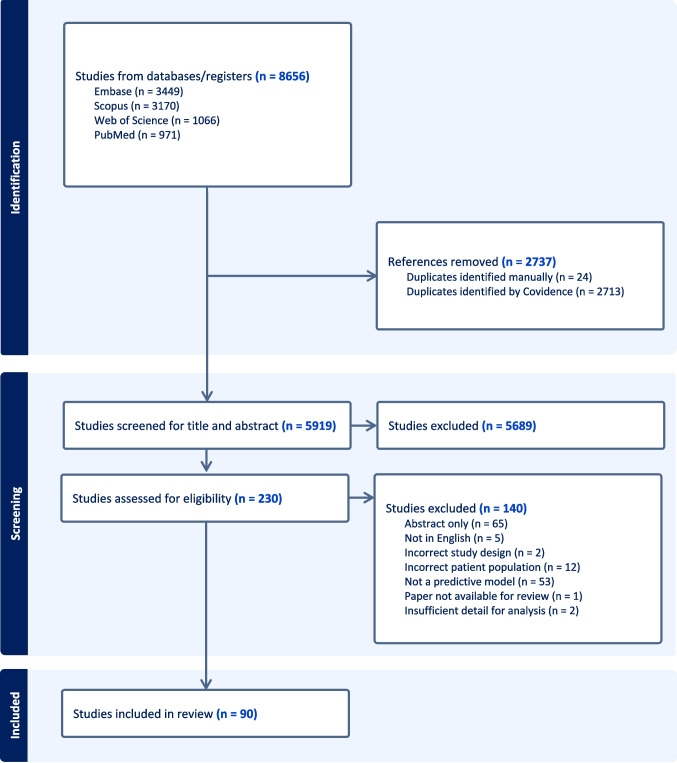
PRISMA diagram for search outcomes and study inclusion

A total of 8656 studies were retrieved from the database search, with 5919 studies after duplicates were removed and 5689 studies excluded in title and abstract screening (Fig. 1). Two hundred thirty studies were reviewed in full, and 90 studies met inclusion criteria (Supplementary Table 1) [9–98].

### Study demographics and cohorts

Of the 90 included studies, 31 (34%) were prospective, 54 (60%) were retrospective, 3 (3%) were a combination of these, and 2 (2%) were cross-sectional. Twenty-five studies predicted FN, and 65 (72%) predicted FN outcomes, of which 28 predicted mortality, 31 predicted microbiological outcomes, 35 predicted complications, and 3 predicted other outcomes of interest. In terms of predictor variables, 59 (66%) used clinical predictor variables, 81 (90%) used laboratory or pathological variables, and 1 used microbiological variables. There were 27 countries represented by the studies, of which most studies came from the USA (13) and South Korea (9). Two studies were multinational.

### Study outcomes—FN prediction

The FN predictive models that have had the most external validation are the Febrile Neutropenia after Chemotherapy (FENCE) and Cycle-Specific Risk of Febrile Neutropenia after Chemotherapy (^CSR^FENCE) scores developed by Aagaard et al. [40, 57] (Table 1). The FENCE score was developed in solid cancer or diffuse large B cell lymphoma (DLBCL) patients receiving first-line chemotherapy, where FN was defined as death or blood culture collection within 3 days of an ANC less than 0.5 × 10^9^/L. Here, using age, infective status, biochemistry and cancer diagnosis, stage, and treatment, the FENCE had good discrimination for FN prediction with a Harrel’s C-statistic of 0.79. This was validated by Zatarah et al. [91] in a similar cohort using a different FN definition of an ANC less than 0.5 × 10^9^/L with temperature $\ge$ 38.2 °C, albeit with a worse discriminatory performance. Thungthong et al. [98] prospectively validated the FENCE in a novel cohort of lymphoma patients who all received G-CSF, with similar discrimination results as the Zatarah validation study.

**Table 1:** Summary of extracted data from FN prediction studies. Extracted data was summarized into the below columns, with further detail in Supplementary Table 1. ALC = absolute lymphocyte count, ALT = alanine transaminase, AMC = absolute monocyte count, ANC = absolute neutrophil count, AUROC = area under the receiver operator characteristic curve, ASCT = autologous haematopoietic stem cell transplant, CDI = clinically documented infection, COPD = chronic obstructive pulmonary disease, CRC = colorectal cancer, CRP = C-reactive protein, ECOG = Eastern Cooperative Oncology Group, ED = emergency department, eGFR = estimated glomerular filtration rate, HIV = human immunodeficiency virus, KPS = Karnofsky performance status, NA = not applicable, NHL = non-Hodgkin’s lymphoma, NPV = negative predictive value, NLR = neutrophil to lymphocyte ratio or negative likelihood ratio, OA = osteoarthritis, PCT = procalcitonin, PLR = platelet-to-lymphocyte ratio or positive likelihood ratio, PPV = positive predictive value, sTNFRI = soluble tumor necrosis factor receptor I, WCC = white cell count

Reference	Inclusion criteria	Inclusion number	Predictor variables	Outcome variables	Performance metrics	External validation performed?
FENCE score						
Aagaard et al. 2018 [40]	Solid cancer or DLBCL Standard first-line chemotherapy	11,229	Age, cancer type, cancer stage, chemotherapy type, albumin, eGFR, infection before chemotherapy, bilirubin, CRP	Positive blood culture or death In sensitivity analysis, Temperature $\ge$ 38.0 and neutrophils < 500/µL	Harrell’s C-statistic	No
Zatarah et al. 2022b [91]	Adult Solid tumour or DLBCL Treatment-naïve	918	FENCE score: age, cancer type, cancer stage, chemotherapy type, albumin, eGFR, infection before chemotherapy, bilirubin, CRP	ANC < 500/µL Temperature ≥ 38.2	AUROC	External validation only
Thungthong et al. 2023 [98]	Adult Lymphoma First-line chemotherapy	135	Fence score: age, cancer type, cancer stage, chemotherapy type, albumin, eGFR, infection before chemotherapy, bilirubin, CRP	Blood culture collected or death within 3 days of a neutrophil count less than 500/µL or leukocyte count less than 2000/µL In sensitivity analysis, Temperature ≥ 38.0 and neutrophils < 500/µL	AUROC Sensitivity Specificity NPV NLR PLR Accuracy	External validation only
Aagaard et al. 2020 [57]	Solid cancers treated with chemotherapy Adult	8076	CSR-FENCE: FENCE score, chemotherapy type, concurrent radiotherapy, cycle number, previous FN or neutropenia, G-CSF use	Blood culture collected or death within 3 days of a neutrophil count less than 500/µL or leukocyte count less than 2000/µL	Harrell’s C-statistic	No
Zatarah et al. 2022a [90]	Adult Solid tumour or DLBCL Treatment-naïve	860 patients, 2870 chemotherapy cycles	CSR-FENCE: FENCE score, chemotherapy type, concurrent radiotherapy, cycle number, previous FN or neutropenia, G-CSF use	ANC < 500/µL Temperature ≥ 38.2	AUROC	External validation only
Externally validated multivariate models						
Schwenkglenks et al. 2013 [17]	Age ≥ 18 years NHL Receiving chemotherapy	1829	INC-EU: Age, weight, previous chemotherapy, planned cyclophosphamide dose, planned cytarabine dose, planned etoposide dose, G-CSF use, baseline albumin, albumin missing, recent infection	FN in first cycle and FN in any cycle	Sensitivity Specificity NPV PPV AUC	External validation only
Bozcuk et al. 2015 [25]	Breast, lung, colorectal cancer Chemotherapy Adult	Derivation: 3880 chemotherapy cycles Validation: 960 patients, 1444 cycles	Age, cycle of current chemotherapy, pre-cycle lymphocyte count, previous FN, type of cancer	FN	Concordance index Sensitivity Specificity	No
Li et al. 2018 [45]	Adult NHL, breast, lung, CRC, ovarian or gastric cancer	15,279	Age, GFR, WCC, cancer type, corticosteroid use, RDI ≥ 85%, chemotherapy use, obesity, diabetes, heart failure, COPD, rheumatoid disease, OA, other autoimmune disease, peptic ulcer disease, thyroid disorder, liver disease, HIV, recent dermatologic or mucosal condition; Lyman model	FN 1 st cycle	AUC	External validation of literature model Only
Venäläinen et al. 2022 [89]	Adult Non-haematological cancer	10,473	Breast cancer, sarcoma, neutrophil count, thrombocyte count, use of taxanes, use of taxanes and monoclonal antibodies, use of topoisomerase inhibitors, use of antimetabolites, use of GCSF, relative dose intensity; Li model	Neutropenic infection: ANC < 500/µL and CRP > 10 within 5 days of this FN as defined	AUROC	Yes
Breast cancer multivariate models						
Chen et al. 2014 [19]	Breast Cancer First cycle chemotherapy	428	ANC, ALC, AMC	FN	AUC Sensitivity Specificity FNR FPR PPV NPV	Yes
Pfeil et al. 2014 [22]	Breast cancer Three cycles of FEC chemotherapy followed by three cycles docetaxel or 4–6 cycles FEC	994	Platelet, Hb, ALT, MRP1rs4148350 SNPs, MRP1rs246221 SNPs, FGFR4rs351855 SNPs	FN	AUC	No
Netterberg et al. 2018 [47]	Adult Breast cancer Chemotherapy	49	CRP, IL-6	FN	NA	No
Bone marrow transplant models						
Buchan et al. 2022 [80]	Adult age ≥ 18 AlloHCT or ASCT	81	HRV, TNF-alpha, IL-6, IL-7	FN Infections positive on microbiology or radiographically (independent of whether FN developed or not)	AUC Sensitivity Specificity PPV NPV	No
Carcò et al. 2022 [81]	Age ≥ 18 Myeloma or lymphoma ASCT	49	WCC, CRP	FN	AUC	No
Machine learning models						
Cho et al. 2020 [59]	Age ≥ 18 Breast cancer Received chemotherapy	933	Eosinophil count, CEA, body surface area, lymphocyte count, neutrophil count, WCC, platelets	FN	AUROC Sensitivity Specificity Accuracy PPV NPV	No
Bozcuk et al. 2022 [79]	Adult Solid cancer on chemotherapy	1909 patients, 4728 chemotherapy cycles	ECOG, type of cancer, history of palliative radiotherapy, previous FN, chemotherapy cycle number, regimen risk, place of chemotherapy administration, pre-cycle lymphocyte count	Temperature 38.3 or 38 ≥ 1 h ANC < 500/µL or ANC < 1000/µL predicted decline to < 500/µL	AUROC Sensitivity Specificity	Yes
Other multivariate models						
Gulleen et al. 2020 [61]	Adult Neutropenia	1531	Temperature, heart rate, magnesium, platelets, haemoglobin	FN	AUROC	Yes
Zhu et al. 2022 [92]	Adult First cycle of etoposide chemotherapy Solid tumour or NHL	1554	KPS, Metastatic sites ≥ 3, heart disease, recent surgery, alkylating agents, Bilirubin, lymphocyte count	FN or severe neutropenia	AUROC	No
Univariate models						
Ribeiro et al. 2013 [16]	Age ≥ 18 years Afebrile with no clinical/radiological infection 48 h prior Haematological disorder Neutropenia expected for > 6 days	26	sTNFRI	FN	Sensitivity Specificity NPV PPV Accuracy	No
Kauffmann-Guerrero et al. 2018 [43]	SCLC Treated at single institution	52	CRP and neutrophils	FN	Sensitivity Specificity PPV Wilks’lambda	No
Shimony et al. 2020 [68]	New acute leukaemia Age ≥ 18 during first induction	138	CRP	FN	AUC Sensitivity Specificity	No
Goto et al. 2023 [96]	FEC100, TC, eribulin mesylate, weekly paclitaxel	327	BMI, nutritional index, WCC, ANC, ALC, NLR	FN	AUC Sensitivity Specificity	No
Kubo et al. 2022 [85]	18–74 years old ECOG 0–2 de novo AML	51	D11 c-D-index	ANC < 500/µL Temperature ≥ 38	Sensitivity Specificity	No
Shimanuki et al. 2018 [48]	Adult TPF chemotherapy for head and neck cancer	50	AMC, ANC	FN	Sensitivity Specificity PPV NPV PLR NLR OR AUC	No
Chantharakhit et al. 2020 [58]	Stage I–III breast cancer	339	NLR	FN	AUROC Sensitivity Specificity PPV NPV PLR NLR	No

Aagaard et al. [57] subsequently expanded on their work with the ^CSR^FENCE score for chemotherapy cycles 2–6 of a patient’s treatment, and added chemotherapy selection, cycle number, previous FN or neutropenia, and G-CSF use as variables. FN was again defined as blood culture collection or death within 3 days of an ANC < 0.5 × 10^9^/L, or, if neutrophils were not measured, a leukocyte count $\le$ 2.0 × 10^9^/L. This showed similar discrimination performance to the original FENCE score and was again validated by Zatarah et al. [90] with an FN definition of an ANC less than 0.5 × 10^9^/L with temperature $\ge$ 38.2 °C. This showed good discrimination with an area under the receiver operator characteristic curve (AUROC) of 0.72 for FN prediction.

Another externally validated model was the Impact of Neutropenia in Chemotherapy – European Study Group (INC-EU) model [99], which was published outside of the search time period scope, but was validated by Schwenkglenks et al. [17]. Using a standard FN definition, this model used variables including demographic information, recent infection, G-CSF use, and chemotherapy administration and dosing. This was validated in non-Hodgkin’s lymphoma (NHL) patients receiving chemotherapy but with worse discrimination.

Bozcuk et al. [25] prospectively developed and externally validated a FN prediction model in breast, lung, and colorectal cancer patients, with demographics, chemotherapy cycle, malignancy, and lymphocyte count as variables. The concordance index in external validation cohorts for this model across six centers was 0.85.

Li et al. [45] modelled common comorbidities, chemotherapy dosing, WCC, renal function, and demographic variables for patients with common oncological malignancies or NHL to predict FN, defined by hospital coding. They compared this to a 2011 model by Lyman et al. [100] and found similar performances. This study was validated by Venäläinen et al. [89], who used a least-absolute shrinkage and selection operator (LASSO) to predict FN from malignancy, treatment, and pathological variables with an AUROC of 0.75 in external validation, which was superior to the Li and Lyman models applied to this cohort.

Three multivariate models predicted FN in breast cancer patients. Chen et al. [19] validated a previous model outside of the search year scope (Jenkin’s model [101]) using pre-treatment hematological parameters; however, they found it had predictive value only when absolute monocyte count was added as a variable (AUROC 0.60). Pfeil et al. [22] assessed single nucleotide polymorphisms and blood parameters and also had a modest performance, while Netterberg et al. [47] used time-to-event analysis to combine IL-6 and CRP as predictor variables in a small cohort, showing that rises in these biomarkers occur prior to FN.

Two models predicted FN in bone marrow transplant patients. Buchan et al. [80] prospectively assessed heart rate variability, TNF-$\alpha$, IL-6, and IL-7 to generate a model with an AUROC of 0.87. Carco et al. [81] demonstrated that prolonged FN was predicted better with WCC than CRP.

Two studies used machine learning to predict FN, both in solid cancers. Cho et al. [59] assessed multiple machine learning methods using laboratory values and body surface area to predict FN in breast cancer patients, with an AUROC of 0.908. Bozcuk et al. [79] developed and externally validated a model combining cancer diagnosis, previous FN, chemotherapy cycle, age, performance status, and lymphocyte count, with an external validation AUROC of 0.78 in patients who did not use G-CSF. This was not improved with the application of artificial neural networks (ANNs) in the model.

In the broader oncology population, Gulleen et al. [61] predicted FN in neutropenic patients with vital signs with magnesium, platelets, and hemoglobin with an AUROC of 0.74. Zhu et al. [92] developed a risk-prediction nomogram using performance status, comorbidities, chemotherapy choice, bilirubin, and lymphocyte count to predict FN with an AUROC of 0.9.

Univariate models have also been used to predict FN with variable efficacy. Predictors included soluble TNF-$\alpha$ I receptor [16], CRP [43, 47, 68], BMI [96], c-D-index [85], ANC [43, 48, 96], neutrophil-to-lymphocyte ratio (NLR) [58], absolute monocyte count (AMC) [48], and IL-6 [47].

### Study outcomes—FN outcome prediction

As described, the MASCC [102] and CISNE [26] scores were developed in 2000 and 2015, respectively, and are currently used in clinical practice. They both combine clinical status and comorbidity data to predict risk of poor outcomes in FN. In the studies that have been analyzed, poor outcomes included respiratory failure, renal failure, hypotension, arrhythmias, DIC, death, ICU admission, or other complications deemed to be serious and clinically significant. However, these outcomes were variably defined between studies. In this review period, 23 studies validated either the CISNE, MASCC, or both, with variable outcomes (MASCC AUROC 0.658–0.768, CISNE AUROC 0.64–0.868 [9, 10, 18, 20, 21, 24, 31–33, 35, 38, 39, 41, 46, 65, 67, 70–72, 74, 82, 86, 93]), which was the recent focus of a systematic review and meta-analysis [8] (Table 2).

**Table 2:** Summary of extracted data from FN outcome prediction models. Extracted data was summarized into the below columns, with further detail in Supplementary Table 1. Serious complications were usually defined as respiratory failure, renal failure, hypotension, altered mental state, confusion, ICU admission, acute heart dysfunction, procedures required to treat febrile illness, DIC, major bleeding, however, see Supplementary Table 1 for further detail as variation was present between studies. The MASCC score includes variables of symptoms, hypotension, COPD, previous fungal infection, dehydration, outpatient status, age. The CISNE score includes variables of ECOG, stress-induced hyperglycemia, COPD, CVD history, mucositis, and monocyte count. The qSOFA score includes variables of mental state, blood pressure, RR. The SOFA score includes variables of SOFA score respiratory status, coagulation, bilirubin, blood pressure, pressor use, GCS and creatinine. AKI = acute kidney injury, ANC = absolute neutrophil count, AMC = absolute monocyte count, Ang-1 = angiotensin 1, Ang-2 = angiotensin 2, AUROC = area under the receiver operator characteristic curve, ASCT = autologous hematopoietic stem cell transplant, CDI = clinically documented infection, cfDNA = cell-free deoxyribonucleic acid, CK-18 = caspase-cleaved cytokeratin-18, COPD = chronic obstructive pulmonary disease, CRP = C-reactive protein, CVD = cardiovascular disease, DIC = disseminated intravascular coagulation, ECOG = Eastern Cooperative Oncology Group, ED = emergency department, ECG = electrocardiogram, eGFR = estimated glomerular filtration rate, FUO = fever of unknown origin, GCS = Glasgow Coma Score, GNB-MDR = gram-negative bacilli multi-drug resistant, HIV = human immunodeficiency virus, HR = heart rate, HSCT = hematopoietic stem cell transplant, ICU = intensive care unit, IFD = invasive fungal disease, IFI = invasive fungal infection, IV = intravenous, JAG1 = jagged 1 protein, LBP = lipopolysaccharide-binding protein, MAPKAPK3 = MAP kinase-activated protein kinase 3, MDI = microbiologically documented infection, MPV = mean platelet volume, MTC = major transplant-related complications, NA = not applicable, NHL = non-Hodgkin’s lymphoma, NPV = negative predictive value, NLR = neutrophil to lymphocyte ratio, PCT = procalcitonin, PLR = platelet-to-lymphocyte ratio or positive likelihood ratio, PPV = positive predictive value, PSPN = presepsin, qSOFA = quick Sequential Organ Failure Assessment, RR = respiratory rate, SFlt-1 = soluble FMS-like tyrosine kinase 1, SIRS = systemic inflammatory response syndrome, SOFA = Sequential Organ Failure Assessment, suPAR = soluble urokinase plasminogen activator receptor, UTI = urinary tract infection, VEGF-A = vascular endothelial growth factor A

Reference	Inclusion criteria	Inclusion number	Predictor variables	Outcome variables	Performance metrics	External validation performed?
MASCC, CISNE and other existing clinical scores						
Ahn et al. 2013 [9]	Adult ED presentation Malignancy FN	355 patients, 400 events	MASCC score, PCT	Bacteremia Shock	AUROC Sensitivity Specificity PPV NPV Accuracy	Yes
Gunderson et al. 2013 [10]	Gynecological malignancy FN with ICD-9 code	83 patients, 91 events	MASCC score	Serious complications Mortality	PPV NPV	External validation only
Carmona-Bayonas et al. 2014 [18]	Adult Oncology ward admission FN Apparently stable patients	692	MASCC score	Serious complications	AUC Sensitivity Specificity	External validation only
Gunalp et al. 2014 [20]	Adult ED presentation FN Chemotherapy	Not stated	Complications: platelets, eGFR, protein, CRP, MASCC Death: Platelets, pulmonary infiltration, protein, RR, MASCC	Serious complications Mortality	Sensitivity Specificity PPV NPV	No
Patil et al. 2014 [21]	FN	91	MASCC score	Mortality	Sensitivity Specificity PPV NPV	External validation only
Bitar et al. 2015 [24]	Age ≥ 18 years FN Antimicrobials	198	MASCC score	Serious complications	Sensitivity Specificity PPV NPV	External validation of literature model only
Carmona-Bayonas et al. 2015 [26]	Age ≥ 18 years FN Mild-or-moderate intensity chemotherapy Outpatients	1133	MASCC score, CISNE score	Serious complications	AUC	External validation of literature model only
Coyne et al. 2017 [33]	Age ≥ 16 Chemotherapy-related FN	230	MASCC score, CISNE score	Serious complications Mortality ICU Bacteremia	Sensitivity Specificity PPV NPV PLR NLR	External validation only
Taj et al. 2017 [38]	Hematological malignancy FN	226	MASCC score	Serious complications Mortality	Sensitivity Specificity PPV	External validation only
Wang et al. 2017 [39]	Age ≥ 16 years Solid tumor or lymphoma FN	120	MASCC score, malaise subscale	Serious complications	Sensitivity Specificity PPV NPV Misclassification AUC	External validation of literature model only
Ahn et al. 2018 [41]	Age > 18 Malignancy FN Emergency department	571	MASCC score, CISNE score	Serious complications Mortality	AUC Sensitivity Specificity PPV NPV PLR NLR	External validation only
Moon et al. 2018 [46]	Age ≥ 18 years Chemotherapy within 30 days Stable patients (for CISNE) FN ED presentation	400	MASCC score, CISNE score	Serious complications Bacteremia	AUC C-index	External validation only
Mohindra et al. 2020 [65]	Age ≥ 12 years Malignancy Chemotherapy-related FN	129	MASCC score, CISNE score	Mortality	AUC Sensitivity Specificity PPV NPV PLR NLR	External validation only
Peyrony et al. 2020 [67]	FN Age ≥ 18 years	249	MASCC score	Inpatient mortality Serious complications	AUC Sensitivity Specificity PPV NPV	External validation only
Bhardwaj et al. 2021 [70]	Age ≥ 18 Inpatient Cancer FN	193	MASCC score	Inpatient death, ICU	AUC	External validation only
Chaftari et al. 2021 [72]	Cancer ED presentation FN Serum lactate and PCT measured at presentation	550	MASCC, lactate, PCT	Mortality Bacteremia Length of Stay	AUROC Specificity Sensitivity NPV PPV	External validation of literature model only
Monuszko et al. 2021 [74]	Gynecological malignancy	50	MASCC score, CISNE score	Serious complications	Sensitivity Specificity PPV NPV	External validation only
García de Guadiana-Romualdo et al. 2019 [49]	Adult Outpatients Chemotherapy for malignancy within 5 weeks prior to ED presentation	111 episodes, 102 patients	MASCC score, PCT, lipopolysaccharide binding protein	Serious complications Bacteremia	AUROC	External validation of literature model only
Matsumoto et al. 2013 [14]	Lung cancer FN	60	MASCC score, CRP	Failure of antimicrobial therapy—five days without fever or serious medical complication	Sensitivity Specificity PPV NPV	External validation of literature model only
Kelly et al. 2018 [44]	FN	39	MASCC 5 metabolic variables RAD18, MAPKAPK3, JAG1	Bacteremia	AUROC	External validation of literature model only
Reyes Mondragón et al. 2021 [76]	Hematological malignancy FN Recent arrival to ED	81 episodes, 72 patients	PCT, compared to MASCC	Death as inpatient Sepsis/Septic Shock	AUROC	External validation of literature model only
Ono et al. 2022 [86]	Lung cancer FN	49	MASCC and CISNE scores	Failure to resume chemotherapy treatment	Sensitivity Specificity Younden’s index	External validation only
Luz Fiuza et al. 2013 [12]	HSCT/Hematology FN	99	MASCC score, CRP, SOFA, VEGF-A, SFlt-1, Ang-1, Ang-2, Ang-2/Ang-1 ratio	Septic shock	AUC	External validation of literature model only
Lappalainen et al. 2020 [63]	Age ≥ 18 AML receiving chemotherapy FN	125 patients, 396 treatment episodes	qSOFA	Infectious mortality ICU treatment	Sensitivity Specificity	External validation of literature model only
Barros et al. 2023 [93]	Adult ASCT	309	MASCC, EBMT, qSOFA and combination of these	BSI, admission to ICU, mortality	Sensitivity Specificity NPV PPV	External validation of literature model only
Kim et al. 2017 [35]	Age ≥ 18 years FN Chemotherapy induced neutropenia	615	qSOFA, MASCC score	Mortality ICU admission Sepsis	Sensitivity Specificity PPV NPV LR + LR-	External validation only
Choi et al. 2022 [82]	Age ≥ 18 Malignancy FN	378	qSOFA, MPV	Mortality Serious complications	AUC	External validation of literature model only
Cetintepe et al. 2021 [71]	FN ICU admission Hematology	60	MASCC score SOFA score (respiratory, coagulation, bilirubin, blood pressure, pressor use, GCS, creatinine) qSOFA	Mortality	Sensitivity Specificity	External validation only
Frairia et al. 2023 [95]	Age ≥ 18 years AML FN	630 FN	qSOFA, National Early Warning Score	Mortality during FN Vasopressor requirement SIRS Respiratory failure Ventilation ICU admission	AUROC Odds ratio	No
Kim et al. 2019 [50]	Adult Septic shock ED presentation Chemotherapy-Induced FN Treated with G-CSF	158	APACHE II score and PLR—both as individual models	1-month survival	AUROC Sensitivity Specificity PPV NPV	External validation of literature model only
Yadav et al. 2021 [78]	> 12 years old Hematologic or solid malignancy Chemotherapy-induced FN	100	MASCC score, PCT	Mortality	AUROC	External validation of literature model only
Machine learning						
Du et al. 2020 [60]	Adult Malignancy	126,013	Intubation and mechanical ventilation, respiratory failure, cardiac arrest and ventricular fibrillation, other aftercare, shock Top 5 ridge: other aftercare, respiratory failure, age, respiratory intubation and mechanical ventilation, acute and unspecified renal failure	Inpatient mortality	AUROC Sensitivity Specificity Precision Recall F1-score	No
Garcia-Vidal et al. 2021 [73]	Hematological malignancy FN	3235 episodes, 349 patients	ML model without description of final variables	GNB-MDR positive culture within 24 h of FN onset	AUROC F1 Score Sensitivity Specificity NPV PPV	No
Padmanabhan et al. 2022 [87]	Age ≥ 18 years FN	1166	Sepsis: Age, region, BSI, Treatment phase, Diagnosis, Sex, Line infection, polymicrobial BSI, UTI MDRO: Age, Type BSI, line, diagnosis, treatment phase, region, sex, colitis, skin infection, UTI Death: Age, sepsis, BSI, Diagnosis	Sepsis, MDRO, mortality	Accuracy Recall AUROC	No
Lynn et al. 2013 [13]	Chemotherapy within 5 weeks prior to ED visit ANC < 500/µL Age ≥ 18 FN	81	Latency of first dose of antibiotics, pneumonia, platelet count, comorbidity, HR	Hypotension SBP < 90 mmHg requiring IV fluids or inotropic agents, respiratory distress requiring high flow oxygen or intubation, GCS < 14, new onset arrhythmia requiring intervention, death	AUC	No
Novel multivariate models						
Ahn et al. 2016 [31]	Age ≥ 16 years Hematological or solid tumor malignancy FN	Derivation: 718 Validation: 283	Age, PCT, ECOG, mucositis, blood pressure, respiratory rate MASCC	Serious complications Bacteremia	AUC	External validation of literature model only
Fonseca et al. 2016 [32]	Adult Solid malignancy on mild-moderate intensity chemotherapy	1133	ECOG, COPD, Chronic CVD, mucositis, monocytes, stress-induced hyperglycemia	Serious complications	C-index Sensitivity Specificity PLR NLR PPV NPV	Yes
Sereeaphinan et al. 2021 [77]	Adult Chemotherapy FN	Not stated	Hb, septic shock, AKI, mechanical ventilation	30-day mortality	AUROC	No
Perazzoli et al. 2019 [53]	FN Inpatient Abdominal/anorectal infection site Hematological malignancy	74 episodes, 69 patients	Baseline hematologic diagnosis, neutropenia severity, duration of neutropenia, therapeutic modality, diagnosis of abdominal or anorectal disease	Inpatient mortality	AUROC	No
Erdem et al. 2023 [94]	≥ 16 years FN Bacteremia Initial BSI after hospital admission	4311	HR, qSOFA, appropriateness of antimicrobial treatment, UTI, Gram-positive BSI	Mortality	AUC Sensitivity Specificity	No
Shan et al. 2022 [88]	Adult allo-HSCT Febrile Weekly PCT Complete clinical data and follow up information	219	Diagnosis, disease status, months from diagnosis, MTC, PCT	CDI BSI Mortality	AUROC C-index	No
Shelburne et al. 2014 [23]	Neutropenia Viridans Group Streptococci BSI	Derivation: 569 Validation: 163	Nosocomial onset, beta-lactam prophylaxis, beta-lactam therapy within 30 days	Beta-lactam resistance in VGS BSI	AUC	No
Marini et al. 2015 [29]	Age ≥ 18 Hematology/oncology Positive blood culture for gram-negative rod	247	Clofarabine use in previous 90 days, rituximab use in previous 90 days, antibiotics for ≥ 14 days in the past 90 days, ICU, respiratory source of culture	Piperacillin/tazobactam resistant gram-negative rod BSI	AUC	No
Univariate models						
Meidani et al. 2013 [15]	Age ≥ 14 years FN Referral from cancer center No antibiotics within 12 h of fever	64	CRP, PCT	Sepsis	Sensitivity Specificity	No
Gardciade et al. 2015 [27]	Adult Chemotherapy-associated FN Solid or hematological tumors Admitted to ED	61 episodes 58 patients	CRP, PCT, IL-6, LBP	CDI or MDI	Sensitivity Specificity PPV NPV PLR NLR AUC	No
Liu et al. 2015 [28]	NHL Chemotherapy Age ≥ 16 years FN CRP and PCT within 48 h	212 episodes; 199 patients	PCT, CRP	BSI CDI Mortality ICU admission	Sensitivity Specificity PPV NPV AUC	No
Michel et al. 2017 [37]	BMT with high-dose chemotherapy	44	PCT, CRP, sTREM-1, IL-8	Serious complications	Sensitivity Specificity AUC	No
Intke et al. 2018 [42]	Adult Inpatients FN Intensive chemotherapy	86	IL1-Ra, CRP, PCT	Severe sepsis	Sensitivity Specificity PPV NPV ROC Younden’s index	No
Kostic et al. 2019 [51]	Admission for high-dose chemotherapy or ASCT FN Adult	28	Presepsin, PCT, IL-8, CRP	Bacteremia	AUROC Sensitivity Specificity PPV NPV Accuracy	No
Shilpakar et al. 2019 [54]	Adult AML or ALL Chemotherapy FN	Not stated	PCT, CRP	Bacteremia	AUROC Sensitivity Specificity	No
Verlinden et al. 2019 [55]	Acute leukemia or stem cell transplant Chemotherapy	121	CRP and PCT	MDI, CDI, IFD, FUO	Sensitivity Specificity PPV NPV Efficiency	No
Yang et al. 2019 [56]	Hematological malignancy Febrile episode PCT, CRP, and serial blood cultures collected	273 episodes	PCT, CRP	Bacteremia	AUROC	No
Halder et al. 2020 [62]	Age 1–60 years Hematological malignancy ANC < 500/µL FN	52 episodes, 50 patients	CRP and PCT	IFI, MDI	AUROC Sensitivity Specificity PPV NPV	No
Odemis et al. 2020 [66]	Age ≥ 18 years Infectious diseases, clinical microbiology or hematology service Case–control—FN or severe neutropenia without fever	88	CRP, PCT, lactate, MCP-1	BSI	AUROC	No
Moustafa et al. 2021 [75]	≥ 14 years old FN Acute leukemia	60	PSPN, PCT, CRP	CDI or MDI	AUROC Specificity Sensitivity NPV PPV	No
Luo et al. 2019 [52]	Hematological disease FN Age ≥ 14 Simultaneous collection of BCs and PCT	1466	PCT	Bacteremia Gram-negative bacteremia Multidrug resistant gram-negative bacteremia	AUROC Sensitivity Specificity	No
Marín et al. 2020 [64]	Critically ill cancer patients ICU admission FN Suspected infection	117	PCT	Positive blood culture Positive gram-negative blood culture	AUROC Sensitivity Specificity PPV NPV	No
Coyne et al. 2022 [83]	Adult FN	198	PCT	Mortality ICU admission	Odds ratio Sensitivity Specificity NPV PPV	No
Kaya et al. 2013 [11]	FN Hematological malignancy	40	suPAR	CDI	Sensitivity Specificity NPV PPV AUC	No
Purhonen et al. 2015 [30]	Adult AML or HSCT treated on the ward FN	100	cfDNA, cfDNA/lymphocyte ratio	Sepsis or septic shock	AUC	No
Efe İris et al. 2017 [34]	Age ≥ 18 FN during chemotherapy Hematological malignancy	31	CD64 on neutrophils	Bacteremia	AUC Sensitivity Specificity PPV NPV	No
Korpelainen et al. 2017 [36]	Adult AML or HSCT FN	87	sCD14	Septic shock, positive blood culture	AUC	No
Alshari et al. 2021 [69]	FN Receiving chemotherapy Treated with G-CSF	80	AMC	ANC difference from baseline D1-D6 Mortality or recurrence of FN	AUROC Sensitivity Specificity	No
Intke et al. 2022 [84]	Adult FN AML post chemotherapy or ASCT	86	CK-18	Severe sepsis, septic shock	AUC	No
Rattanathammethee et al. 2023 [97]	Aged ≥ 18 New diagnosis AML First induction 7 + 3 chemotherapy Adequate serial CBC during admission	101	c-D-index, D-index, duration of grade 4 neutropenia, duration of profound neutropenia	IFI	Sensitivity Specificity PPV NPV ROC	No

Several studies combined the MASCC with other variables, including Gunalp et al. [20] who developed a novel model using inflammatory markers and laboratory values with the MASCC to predict mortality or poor outcomes. Ahn et al. [9] added procalcitonin and clinical factors with the MASCC to predict poor outcomes or bacteremia, which was superior to the MASCC for predicting both outcomes. Others have combined MASCC with single variables to improve performance including lipopolysaccharide binding protein [49], CRP [14], and patient-reported outcomes [39]. Kelly et al. [44] compared the MASCC with metabolic and gene expression profiles to predict bacteremia in FN patients, where the omics model outperformed the MASCC. Finally, others have compared univariate models with the MASCC for single outcome measures and found them superior to the MASCC, including for PCT and bacteremia [72], PCT and mortality [76, 78], and angiopoietin-12/angiopoietin-1 ratio for predicting septic shock [12].

The SOFA, qSOFA, APACHE II, and National Early Warning Scores (NEWS) are used in sepsis prediction and have also been applied to FN outcome prediction. Barros et al. [93] showed no clear superiority between the MASCC, European Society for Bone Marrow Transplantation score (EBMT), or qSOFA in predicting complicated FN or mortality in BMT patients in their cohort. Kim et al. [35] demonstrated that in predicting sepsis, mortality, and ICU admission, the MASCC was superior to the qSOFA. Choi et al. [82] showed that combining the qSOFA with mean platelet volume was inferior to the MASCC for predicting complicated FN. Cetintepe et al. [71] showed that the qSOFA, SOFA, and MASCC all performed poorly in FN patients in ICU for predicting mortality; here, the MASCC was sensitive but not specific (0.188 and 0.75, respectively) and the qSOFA and SOFA was specific (0.917 and 0.833, respectively) but not sensitive (0.00 each). Frairia et al. [95] compared qSOFA and NEWS and found that both predicted mortality in acute myeloid leukemia (AML) patients with FN (AUROC 0.984 and 0.969). Kim et al. [50] demonstrated that the APACHE II score outperformed the platelet-to-lymphocyte ratio (PLR) for predicting 30-day mortality (AUROC 0.730 and 0.666, respectively).

In terms of novel multivariate or machine learning models not currently in clinical use, five predict mortality, five predict microbiological complications, and one predicts serious complications. Fonseca et al. [32] developed and externally validated a nomogram using clinical parameters to predict serious complications in seemingly stable solid organ malignancy FN patients. Perazzoli et al. [53] predicted inpatient mortality in hematology and BMT patients with FN using neutropenia severity, therapeutic regimen, and malignancy diagnosis as variables, with an AUROC of 0.82. Du et al. [60] compared multiple machine learning models to predict mortality using a database of 126,013 admissions, of which gradient boosting tree was the superior modelling technique, but all models had an AUROC of 0.92. This was performed including variables such as cardiac arrest, mechanical ventilation, and respiratory failure. Sereeaphinan et al. [77] predicted mortality in hematology and oncology patients using hemoglobin concentration, septic shock, mechanical ventilation, and serum creatinine, with an AUROC of 0.89. Erdem et al. [94] predicted mortality in FN patients with bacteremia, with clinical and microbiological variables.

With respects to microbiological complication prediction in FN, Shan et al. [88] predicted both mortality and bacteremia with in allogenic stem cell transplant recipients, using malignancy variables, major transplant complications, and procalcitonin, with improved prediction for bacteremia compared with mortality. Shelburne et al. [23] predicted beta-lactam resistance in viridans group streptococci bacteremia in FN patients based on antimicrobial use and nosocomial onset with very high discrimination. Similarly, Marini et al. [29] predicted piperacillin/tazobactam resistance in gram-negative rod bacteremia for FN patients on the basis of antibiotic use, chemotherapy regimen, source of positive culture, and ICU admission, with an AUROC of 0.894. Garcia-Vidal et al. [73] used machine learning methods to predict multidrug-resistant gram-negative bacilli bacteremia with an AUROC of 0.7945, but a sensitivity of 0.4895, likely representing the low frequency of events. Padmanabhan et al. [87] predicted multiple clinical endpoints with machine learning with very good discrimination, including sepsis, multi-drug-resistant bacteremia, and mortality. These models used variable combinations of age, malignancy, and infection type. Lynn et al. [13] used machine learning to predict complications in FN using time to antibiotics, pneumonia, heart rate, and platelet count.

Twenty-six studies used univariate models to predict FN outcomes with variable cutoffs and predictive power. These include CRP [11, 15, 27, 28, 36, 37, 42, 51, 55, 56, 62, 66, 84], PCT [11, 15, 27, 28, 36, 37, 42, 51, 52, 55, 56, 62, 64, 66, 75, 84], interleukin-1 receptor antagonist [42], interleukin-6 [27], interleukin-8 [37, 51], soluble CD-14 [36], lipopolysaccharide binding protein [27], PLR [50], neutrophil CD64 expression [34], sTREM-1 [37], lactate [66], presepsin [51], monocyte chemoattractant protein-1 [66], monocyte count [69], D-index [97], caspase-cleaved cytokeratin-18 [84], cell-free DNA [30], and serum urokinase plasminogen activation receptor [11].

### Risk of bias

Overall, all 90 studies were rated high risk of bias using the PROBAST tool (Supplementary Table 2). Within the specific domains, 28 (38%) were high risk in the participants domain, 13 (18%) were high risk in the predictors domain, 46 (62%) were high risk in the outcomes domain, and all 90 were high risk in the analysis domain. The most common source of risk of bias within the analysis domain was model discrimination and calibration assessment (80 studies).

## Discussion

FN is a life-threatening complication of therapy within cancer care, and reliably predicting its occurrence and the risk of serious complications is important. This systematic review has summarized and critically appraised the last decade of research predicting FN and its outcomes and has revealed significant heterogeneity in both predictor variables and outcomes used in model development. Additionally, the risk of bias within model development, lack of external validation, and reduced discriminatory power when external validation is performed limits utility and translation of these models in real-world practice, despite evidence of promising discriminatory ability within each individual study’s derivation cohorts.

Clinically, there are multiple decision-making scenarios that these tools can potentially assist in. When selecting therapeutic regimens, including chemotherapy dosing, clinicians weigh the risks of complications against the potential benefit of treatment. They can also opt to include prophylactic G-CSF or antimicrobials in the treatment regimen to prevent infective complications, including FN. At the time of FN diagnosis, the decision to treat as an inpatient or outpatient, choice of antimicrobial agents and further supportive measures, as well as need for high-dependency care, may all depend on patient risk of deterioration, microbiological complications or mortality. These models could be invaluable in the clinical decision-making process for each of these considerations. Unfortunately, a previous systematic review and meta-analyses demonstrated the clinical inadequacy in FN outcome prediction of the MASCC and CISNE, where the MASCC had a pooled a sensitivity and specificity of 55.6% and 86.0%, respectively, for the prediction of serious complications in FN patients, and the CISNE had a sensitivity and specificity of 78.9% and 64.9%, respectively [8]. These findings were supported by this review; however, this review also incorporated the many studies that applied these models in novel cohorts, such as patients with leukemia, and for novel outcomes, such as mortality or bacteremia only instead of all complications. This heterogeneity limited an appropriate meta-analysis from being performed as part of this review. Similarly, univariate pathological variables such as PCT, CRP, and IL-6 have been previously found to not have sufficient discrimination ability alone in meta-analysis [103], though the current review did find models frequently using these variables in multivariate modelling, where they may find future utility. There are also significant limitations in discriminatory power in the novel multivariate models, especially when they are externally validated. When applied to novel cohorts in validation studies, the FENCE/^CSR^FENCE scores, the Li model, Lyman model, and Jenkin’s model underperform their derivation studies, a well-established phenomenon that is often a consequence of a model “overfitting” an individual cohort. Although the Bozcuk model was a multinational study that showed promise in their same-study external validation cohort, this has yet to be validated in additional studies.

The existing model limitations are compounded by their ROB. Although all studies were rated as high risk of bias, there were differences between studies as to how many and which domains conferred this risk. The most common ROB source was in the data analysis—specifically, where very few models have appropriate calibration, which is vital to the translatability and generalizability of models developed from single cohorts. Studies often also do not cross-validate their model to optimize the internal validation of a chosen model, further compounding this risk. Another common source of ROB in these studies was variability in definitions of fever and neutropenia, sometimes between validation and model development studies, which prevents direct cohort comparison. Guideline definitions [3] should be used where possible to afford direct model comparison and maximize clinical translation. Within the population definition, G-CSF use and prophylactic antimicrobial use was often not communicated in study methods, which makes the applicability of the model in specific cohorts difficult to ascertain. Separately, having these as predictive variables when they are likely one of the desired interventions for patients identified as high risk by the model further limits model utility. Intra-cohort variability can also confound the discriminatory power of a model, such as when hematology and oncology patients are included in a single model, and when there is a mix of G-CSF and antimicrobial prophylaxis use within one modelled cohort. These factors should be homogenized where possible in model development cohorts.

There were a variety of predictor variables used in the assessed models. Broadly, these included demographic and clinical variables such as patient age, malignancy type, chemotherapeutic agents, and vital signs; common laboratory variables such inflammatory markers, cell counts and biochemistry; and microbiological variables, such as source of infection and BSI species. Although most models used some combination of laboratory and clinical variables, the heterogeneity between models prevents any broad conclusions to be drawn about the likely utility of any one predictor variable within a multivariate model. These broad domains, in combination with the biomarkers identified in the univariate models discussed, should form the basis for future models.

Machine learning models represent a small fraction of the studies within this review period, with four studies each predicting FN and FN outcomes. As recently reviewed by Gallardo-Pizarro et al. [104], machine learning has the benefit of using large numbers of variables that can identify and use subtle relationships not detectable by traditional modelling techniques, but with a potentially increased risk of overfitting the dataset [105]. These models are, however, very sensitive to the quality of the input data in model development and can have their uptake limited by clinician unease with the opaque nature of how the algorithm arrives at a result, the so-called black box phenomenon. In this review, none of the machine learning models were externally validated, which limits commentary on their clinical applicability and discriminatory ability. However, the results from these approaches are promising, and it has been well established that ML more broadly clearly has the ability to produce powerful predictions that outperforms traditional modelling methods. Furthermore, with a growing need for integrated digital workflows within healthcare and increasing dataset sizes and complexity, integrating well-validated machine learning models into EMR workflows may be the best path forward. This integration would allow real-time monitoring and prediction of both FN and its outcomes, which, with early intervention and clinician notification, could also further improve patient outcomes. EMR integration approaches have been assessed in other clinical domains, such as in acute kidney injury management [106] and glycemic control monitoring [107], which provides a framework for the field to consider this approach. In addition, ML integration may also afford self-supervised improvement overtime when integrated into an EMR [108, 109], which has the benefit of incorporating re-training to continue to improve prediction as a population and data changes. As there are barriers at both a systems level and a clinician level to the integration and uptake of these models in all fields, any future developments toward integrated models will need consultation with key stakeholders and training to maximize their utility. However, these future approaches require robust models to be developed and validated first, which should be the concurrent focus of the field.

The strengths of this review were the robust and well-validated ROB assessment, the broad search strategy used to encapsulate existing and novel models, and the granularity of detail pertaining to each study that was extracted. This study was limited by the absence of a meta-analysis, which was not performed due to the breadth and heterogeneity of study prediction variables and outcomes. Further limitations were that only studies from the search strategy and date range were included, which may have resulted in the inadvertent exclusion of studies of interest.

## Conclusion

This study has systematically reviewed and critically appraised the predictive models used to predict FN and its outcomes over the past decade. Although these models do show promise and can have great clinical benefit once translatable, the current models that have been developed have significant ROB and limited external validation. Future model development should aim to use guideline-accepted FN definitions and should use multivariate or machine learning modelling that combines both commonly collected clinical, laboratory, and microbiological predictor variables with novel biomarkers. Prospectively externally validating promising models should continue to be a focus of the field. Novel model development may benefit from further exploration of machine learning methods, and all models should have the ultimate goal of translating into clinical practice, which may be best achieved through incorporation into existing workflows, such as EMR.

## Supplementary Information

Below is the link to the electronic supplementary material.

ESM 1 (DOCX 81.2 KB)

ESM 2 (DOCX 37.1 KB)

ESM 3 (DOCX 16.3 KB)

## Data Availability

Data is provided within the manuscript or supplementary information files.
