# Supplementary material for: Clinical prediction models for febrile neutropenia and its outcomes: a systematic review
Source: Support Care Cancer. 2025 Jun 4;33(7):537. doi: 10.1007/s00520-025-09562-y (PMC12137469; doi:10.1007/s00520-025-09562-y)
Supplement: Supplementary file 1 — (DOCX 81.2 KB) [file 520_2025_9562_MOESM1_ESM.docx]

**Supplementary Table 1: Summary of Extracted Data from Included Studies**AKI = acute kidney injury, ALC = absolute lymphocyte count, ALT = alanine transaminase, AMC = absolute monocyte count, ANC = absolute neutrophil count, Ang-1 = angiotensin 1, Ang-2 = angiotensin 2,AUROC = area under the receiver operator characteristic curve, ASCT = autologous haematopoietic stem cell transplant, CCF = congestive cardiac failure, CDI = clinically documented infection, cfDNA = cell-free deoxyribonucleic acid, CK-18 = caspase-cleaved cytokeratin-18, COPD = chronic obstructive pulmonary disease, CRC= colorectal cancer, CRP = C-reactive protein, CVD = cardiovascular disease, DIC = disseminated intravascular coagulation, ECOG = Eastern Cooperative Oncology Group, ED = emergency department, ECG = electrocardiogram, eGFR = estimated glomerular filtration rate, FUO = fever of unknown origin, GCS = Glasgow Coma Score, GNB-MDR = gram-negative bacilli multi-drug resistant, HIV = human immunodeficiency virus, HR = heart rate, HSCT = haematopoietic stem cell transplant, ICU = intensive care unit, IFD = invasive fungal disease, IFI = invasive fungal infection, IV = intravenous, JAG1 = jagged 1 protein, KPS = Karnofsky performance status, LBP = lipopolysaccharide-binding protein, MAPKAPK3 = MAP kinase-activated protein kinase 3, MDI = microbiologically documented infection, MPV = mean platelet volume, MTC = major transplant-related complications, NA = not applicable, NHL = non-Hodgkin’s lymphoma, NPV = negative predictive value, NLR = neutrophil to lymphocyte ratio, OA = osteoarthritis, PCT = procalcitonin, PLR = platelet-to-lymphocyte ratio, PPV = positive predictive value, PSPN = presepsin, qSOFA = quick Sequential Organ Failure Assessment, RR = respiratory rate, SFlt-1 = soluble fms-like tyrosine kinase 1, SIRS = systemic inflammatory response syndrome, SOFA = Sequential Organ Failure Assessment, sTNFRI = Soluble tumor necrosis factor receptor I, suPAR = soluble urokinase plasminogen activator receptor, UTI = urinary tract infection, VEGF-A = Vascular Endothelial Growth Factor A, WCC = white cell count

| **Study Number** | **Reference** | **Population** | | | | | **Prediction** | | | **Endpoint** | | **Analysis** | | | | | | | | | | | |
| --- | --- | --- | --- | --- | --- | --- | --- | --- | --- | --- | --- | --- | --- | --- | --- | --- | --- | --- | --- | --- | --- | --- | --- |
|  |  | **Location** | **Dataset Type** | **Inclusion Criteria** | **FN definition** | **Number** | **Variable Classification** | **Variables in final model** | **Model Type** | **Endpoint Classification** | **Specific Outcome** | **Performance Metrics** | **Internal validation result** | | | | | | | **External validation** | | | **Calibration Performed?** |
| 1 | Ahn et al 2013[9] | Asan Medical Center, South Korea | Single Centre | Adult  ED presentation Malignancy FN | Temperature ≥ 38°C ANC < 1500/uL | 355 patients, 400 events | Clinical, pathological | MASCC score (symptoms, hypotension, COPD, previous fungal infection, dehydration, outpatient status, age), PCT | Multivariate | Microbiological, complications | Bacteraemia Shock - hypotension <90mmHg or reduction of 40mmHg from baseline despite fluid resuscitation | AUROC Sensitivity Specificity PPV NPV Accuracy | PCT bacteraemia: 0.815 0.71 0.82 0.30 0.96 0.81 | | | | | PCT shock: 0.916 0.84 0.90 0.40 0.99 0.90 | | MASCC Bacteremia 0.815 0.46 0.90 0.33 0.94 0.85 | | MASCC shock 0.909 0.68 0.90 0.35 0.97 0.89 | Not performed |
| 2 | Gunderson et al 2013[10] | University of Oklahoma Health Sciences Center, USA | Single Centre | Gynecological malignancy FN with ICD-9 code | Temperature ≥38.3°C or ≥38°C ≥ 1 hour ANC < 1500/uL | 83 patients, 91 events | Clinical, pathological | MASCC score (symptoms, hypotension, COPD, previous fungal infection, dehydration, outpatient status, age) | Multivariate | Complications | Serious complications defined as cardiac failure, respiratory failure, renal failure, hypotension, altered mental state, ICU admission, arrhythmias, ECG changes, fungal infection, allergic reaction Mortality within 14 days | PPV NPV | NA | | | | | | | 0.50 0.90 | | | Not performed |
| 3 | Kaya et al 2013[11] | Karadeniz Technical University Hospital, Turkey | Single Centre | FN Haematological malignancy | Not stated | 40 | Pathological | suPAR | Univariate | Microbiological | Documented infection - bacteraemia, pneumonia, UTI | Sensitivity Specificity NPV PPV AUC | suPAR: 1.00 0.69 1.00 0.70 0.81 | | | | | | | NA | | | Not performed |
| 4 | Luz Fiuza et al 2013[12] | University of Campinas, Brazil | Single Centre | HSCT/ Haematology FN | Temperature ≥38°C ANC<500/uL | 99 | Clinical, pathological | MASCC score (symptoms, hypotension, COPD, previous fungal infection, dehydration, outpatient status, age), CRP, SOFA, VEGF-A, SFlt-1, Ang-1, Ang-2, Ang-2/Ang-1 ratio | Univariate | Complications | Septic shock | AUC | Ang-2/Ang-1 ratio 0.68 | | | | | | | MASCC  0.59 | | | Not performed |
| 5 | Lynn et al 2013[13] | Single centre Taoyuan, Taiwan | Single Centre | Chemotherapy within 5 weeks prior to ED visit ANC <500/ul Age ≥18 FN | Temperature ≥38.3°C within 24 hours of ANC <500/uL | 81 | Clinical, pathological | Latency of first dose of antibiotics, pneumonia, platelet count, comorbidity, HR | ML | Complications | Hypotension SBP <90mmHg requiring IV fluids or inotropic agents, respiratory distress requiring high flow oxygen or intubation, GCS <14, new onset arrhythmia requiring intervention, death | AUC | 0.779 | | | | | | | NA | | | Not performed |
| 6 | Matsumoto et al 2013[14] | Single centre, Japan | Single Centre | Lung cancer FN | Temperature ≥37.5°C ANC <1000/ul | 60 | Clinical, pathological | MASCC score (symptoms, hypotension, COPD, previous fungal infection, dehydration, outpatient status, age), CRP | Multivariate | Complications | Failure of antimicrobial therapy - five days without fever or serious medical complication | Sensitivity Specificity PPV NPV | MASCC + CRP 0.870 0.462 0.851 0.50 | | | | | | | MASCC 0.695 0.385 0.800 0.26 | | | Not performed |
| 7 | Meidani et al 2013[15] | Single Centre, Iran | Single Centre | Age ≥ 14 years FN Referral from cancer centre No antibiotics within 12 hours of fever | Temperature ≥38.3°C or ≥38°C ≥ 1 hour ANC < 500/uL or ANC <1000/uL predicted decline to <500/uL | 64 | Pathological | CRP, PCT | Univariate | Complication | Sepsis - suspected or proven infection with SIRS | Sensitivity Specificity | PCT: 92.5 97.3 | | | | | | | NA | | | Not performed |
| 8 | Ribeiro et al 2013[16] | Single centre, Brazil | Single Centre | Age ≥ 18 years Afebrile with no clinical/ radiological infection 48 hours prior Haematological disorder Neutropenia expected for > 6 days | Temperature ≥38.3°C or ≥37.8°C ≥ 1 hour ANC <1000/uL | 26 | Pathological | sTNFRI | Univariate | FN | FN | Sensitivity Specificity NPV PPV Accuracy | 0.65 0.87 0.93 0.46 0.70 | | | | | | | NA | | | Not performed |
| 9 | Schwenkglenks et al 2013[17] | Multicentre, 15 countries | Multicentre/ Database | Age ≥ 18 years  NHL Receiving chemotherapy | Temperature ≥ ≥38°C ANC < 500/uL or ANC <1000/uL predicted to decrease to < 500/uL | 1829 | Clinical, pathological | Age, weight, previous chemotherapy, planned cyclophosphamide dose, planned cytarabine dose, planned etoposide dose, G-CSF use, baseline albumin, albumin missing, recent infection | Multivariate | FN | FN in first cycle and FN in any cycle | Sensitivity Specificity NPV PPV AUC | NA | | | | | | | First cycle: 0.59 0.59 0.95 0.10 0.64 | Any cycle: 0.66 0.67 0.90 0.30 0.71 | | Not performed |
| 10 | Carmona-Bayonas et al 2014[18] | Tertiary Centre, Spain | Single Centre | Adult Oncology ward admission FN Apparently stable patients | Temperature ≥38.3°C or ≥38°C ≥ 1 hour ANC < 500/uL or ANC <1000/uL with prediction to be less than <500/uL | 692 | Clinical | MASCC score (symptoms, hypotension, COPD, previous fungal infection, dehydration, outpatient status, age) | Multivariate | Complications | Serious complications defined as hypotension, respiratory failure, ICU admission, disseminated intravascular coagulation, confusion or altered mental state, CCF, bleeding requiring transfusion, arrhythmia requiring treatment, renal failure | AUC Sensitivity Specificity | NA | | | | | | | 0.74 0.36 0.94 | | | Not performed |
| 11 | Chen et al 2014[19] | Sun Yat-sen University Cancer Center | Single Centre | Breast Cancer First cycle chemotherapy | Temperature ≥38.5°C ANC < 500/uL or ANC <1000/uL with prediction to be less than <500/uL | 428 | Pathological | ANC, ALC, AMC | Multivariate | FN | FN | AUC Sensitivity Specificity FNR FPR PPV NPV | 0.60 0.382 0.812 0.618 0.188 0.231 0.891 | | | | | | | Validated model groups - no significant difference | | | Not performed |
| 12 | Gunalp et al 2014[20] | University of Ankara Hospital, Turkey | Single Centre | Adult ED presentation FN Chemotherapy | Temperature ≥38.3°C or ≥38°C ≥ 1 hour ANC < 500/uL | Not stated | Clinical, pathological | Complications: platelets, eGFR, protein, CRP, MASCC Death: Platelets, pulmonary infiltration, protein, RR, MASCC | Multivariate | Mortality, complications | Respiratory failure, severe bleeding, ICU, renal failure, fungal infection, refractory hypotension, altered mental status, arrythmia requiring treatment, chronic heart failure, allergic reactions, mortality | Sensitivity Specificity PPV NPV | Mortality: 0.75 0.89 0.77 0.88 | | | | | Complication: 0.81 0.78 0.81 0.79 | | NA | | | Not performed |
| 13 | Patil et al 2014[21] | Malabar Cancer Center, India | Single Centre | FN | Not stated | 91 | Clinical | MASCC score (symptoms, hypotension, COPD, previous fungal infection, dehydration, outpatient status, age) | Multivariate | Mortality | Mortality | Sensitivity Specificity PPV NPV | NA | | | | | | | 0.857 0.75 0.984 0.222 | | | Not performed |
| 14 | Pfeil et al 2014[22] | University Hospitals Leuven, Belgium | Single Centre | Breast cancer Three cycles of FEC chemotherapy followed by three cycles docetaxel or 4-6 cycles FEC | Temperature ≥38°C  ANC < 500/uL | 994 | Pathological | Platelet, Hb, ALT, MRP1rs4148350 SNPs, MRP1rs246221 SNPs, FGFR4rs351855 SNPs | Multivariate | FN | FN | AUC | 0.661 | | | | | | | NA | | | Yes |
| 15 | Shelburne et al 2014[23] | MD Anderson Cancer Center, USA | Single Centre | Neutropenia Viridans Group Streptococci BSI | Temperature ≥38.0°C ANC < 500/uL | Derivation: 569 Validation: 163 | Clinical, pathological | Nosocomial onset, beta-lactam prophylaxis, beta-lactam therapy within 30 days | Multivariate | Microbiological | Beta-lactam resistance in VGS BSI | AUC | 0.9 | | | | | | | NA | | | Not performed |
| 16 | Bitar et al 2015[24] | Four centres, USA | Multi-centre | Age ≥ 18 years FN Had received antimicrobial therapy for neutropenia fever | Fever self-reported, diagnosis of fever or fever undefined ANC < 500/uL | 198 | Clinical, pathological | MASCC score (symptoms, hypotension, COPD, previous fungal infection, dehydration, outpatient status, age) | Multivariate | Complications | Serious complications defined as cardiac failure, respiratory failure, renal failure, bleeding requiring transfusion, hypotension, DIC, altered mental state, arrhythmia, ECG changes, other serious complications requiring inpatient admission | Sensitivity Specificity PPV NPV | NA | | | | | | | 0.940 0.296 0.577 0.829 | | | Not performed |
| 17 | Bozcuk et al 2015[25] | Two centres for model development, four centres for validation; Turkey | Multi-centre | Breast, lung, colorectal cancer Chemotherapy Adult | Temperature ≥38.3°C or ≥38°C ≥ 1 hour ANC < 500/uL or ANC <1000/uL predicted decline to <500/uL | Derivation:  3880 chemotherapy cycles  Validation: 960 patients, 1444 cycles | Clinical, pathological | Age, cycle of current chemotherapy, pre-cycle lymphocyte count, previous FN, type of cancer | Multivariate | FN | FN | Concordance index Sensitivity Specificity | 0.95 0.76 0.98 | | | | | | | 0.85 Not reported Not reported | | | Not performed |
| 18 | Carmona-Bayonas et al 2015[26] | 24 centres, Spain | Multicentre | Age ≥ 18 years FN Mild-or-moderate intensity chemotherapy Outpatients | Temperature ≥38°C  ANC < 500/uL or ANC <1000/uL predicted decline to <500/uL | 1133 | Clinical, pathological | MASCC score (symptoms, hypotension, COPD, previous fungal infection, dehydration, outpatient status, age), CISNE score (ECOG, stress-induced hyperglycemia, COPD, CVD history, mucositis, monocyte count) | Multivariate | Complications | Serious complications defined as cardiac failure, respiratory failure, renal failure, bleeding requiring transfusion, hypotension, altered mental state, arrhythmia, ECG changes, other serious complications requiring inpatient admission | AUC | NA | | | | | | | CISNE:  0.868  MASCC: 0.768  Talcott: 0.669 | | | Yes |
| 19 | Gardciade et al 2015[27] | University Hospital Santa Lucia, Spain | Single Centre | Adult  Chemotherapy-associated FN Solid or haematological tumours Admitted to ED | Temperature ≥38.3°C or ≥38°C ≥1 hour ANC < 500/uL or ANC <1000/uL predicted decline to <500/uL | 61 episodes 58 patients | Pathological | CRP, PCT, IL-6, LBP | Univariate | Microbiological | Infection: clinically documented or microbiological (BSI or other) | Sensitivity Specificity PPV NPV PLR NLR AUC | PCT: 0.844 0.828 0.844 0.828 4.89 0.19 0.88 | | | | | | | NA | | | Not performed |
| 20 | Liu et al 2015[28] | Sun Yat-sen University Cancer Center | Single Centre | NHL Chemotherapy Age ≥16 years FN CRP and PCT within 48 hours | Temperature ≥38.3°C or ≥38°C ≥1 hour ANC < 500/uL | 212 episodes; 199 patients | Pathological | PCT, CRP | Univariate | Microbiological, Complications, Mortality | Positive blood culture Clinically documented infection Mortality ICU Admission | Sensitivity Specificity PPV NPV AUC | Mortality: 0.778 0.886 0.500 0.965 0.864 | | | | | ICU: 0.913 0.873 0.467 0.988 0.926 | | NA | | | Not performed |
| 21 | Marini et al 2015[29] | University of Michigan Health System, USA | Single Centre | Age ≥18 Haematology/oncology Positive blood culture for gram-negative rod | Temperature ≥38.3°C within 24 hours ANC <500/uL | 247 | Clinical, pathological | Clofarabine use in previous 90 days, rituximab use in previous 90 days, antibiotics for ≥14 days in the past 90 days, ICU, respiratory source of culture | Multivariate | Microbiological | Piperacillin/tazobactam resistant gram-negative rod BSI | AUC | 0.894 | | | | | | | NA | | | Not performed |
| 22 | Purhonen et al 2015[30] | Kuopio University Hospital, Finland | Single Centre | Adult  AML or HSCT treated on the ward FN | Temperature ≥38.3°C or ≥38°C for 1 hour ANC < 500/uL or ANC <1000/uL with prediction to be less than <500/uL | 100 | Pathological | cfDNA, cfDNA/lymphocyte ratio | Univariate | Complication | Sepsis or septic shock | AUC | AML patients: 0.76  Lymphoma patients: 0.77 | | | | | | | NA | | | Not performed |
| 23 | Ahn et al 2016[31] | Asan Medical Center, South Korea | Single Centre | Age ≥ 16 years Haematological or solid tumour malignancy FN | Temperature ≥38.0°C ANC < 500/uL or ANC <1000/uL predicted decline to <500/uL | Derivation: 718 Validation: 283 | Clinical, pathological | Age, PCT, ECOG, mucositis, blood pressure, respiratory rate | Multivariate | Complications, microbiological | Serious complications defined as cardiac failure, respiratory failure, renal failure, hypotension, altered mental state, ICU admission, arrhythmias, death Bacteraemia | AUC | Poor outcome: 0.797  Bacteraemia: 0.867 | | | | | | | MASCC poor outcome: 0.772  MASCC bacteraemia: 0.814 | | | Not performed |
| 24 | Fonseca et al 2016[32] | Multiple centres; Spain | Multi-centre/ Database | Adult Solid malignancy on mild-moderate intensity chemotherapy | Temperature ≥38.0°C ANC < 500/uL or ANC <1000/uL predicted decline to <500/uL | 1133 | Clinical | ECOG, COPD, Chronic CVD, mucositis, monocytes, stress-induced hyperglycemia | Multivariate | Complications | Hypotension, acute renal failure, acute cardiac failure, respiratory failure, arrhythmia, major bleeding, delirium, acute abdomen, DIC | C-index Sensitivity Specificity PLR NLR PPV NPV | 0.855 NA NA NA NA NA NA | | | | | | | 0.831 0.66 0.83 3.88 0.41 0.48 0.91 | | | Yes |
| 25 | Coyne et al 2017[33] | Two centres, not stated | Multicentre | Age ≥ 16 Chemotherapy-related FN | Temperature ≥38°C  ANC < 1000/uL | 230 | Clinical, pathological | MASCC score (symptoms, hypotension, COPD, previous fungal infection, dehydration, outpatient status, age), CISNE score (ECOG, stress-induced hyperglycemia, COPD, CVD, mucositis, monocyte count) | Multivariate | Mortality, microbiological, complications | Serious complications defined as cardiac failure, respiratory failure, renal failure, hypotension, altered mental state, other disease process necessitating change in clinical management Mortality ICU Positive blood culture results | Sensitivity Specificity PPV NPV PLR NLR | NA | | | | | | | CISNE: 0.304 0.983 0.981 0.328 17.9 0.7 | MASCC: 0.830 0.542 0.840 0.525 1.8 0.3 | | Not performed |
| 26 | Efe İris et al 2017[34] | Okmeydani Training and Research Hospital, Turkey | Single Centre | Age ≥ 18 FN during chemotherapy Haematological malignancy | Temperature ≥38.3°C or two consecutive ≥38.0°C ANC < 500/uL or ANC <1000/uL predicted decline to <500/uL | 31 | Pathological | CD64 on neutrophils | Univariate | Microbiological | Bacteraemia | AUC Sensitivity Specificity PPV NPV | 0.866 0.941 0.643 0.762 0.900 | | | | | | | NA | | | Not performed |
| 27 | Kim et al 2017[35] | Asan Medical Center, South Korea | Single Centre | Age ≥ 18 years FN Chemotherapy induced neutropenia | Temperature ≥38.0°C ANC < 500/uL or ANC <1000/uL predicted decline to <500/uL | 615 | Clinical | qSOFA: Confusion, blood pressure, respiratory rate MASCC score (symptoms, hypotension, COPD, previous fungal infection, dehydration, outpatient status, age) | Multivariate | Mortality, Complications | 28 day mortality ICU admission Sepsis defined as "infection, together with its systemic manifestations, as determined using clinical, microbiological, laboratory and radiologic data" | Sensitivity Specificity PPV NPV LR+ LR- | NA | | | | | | | * | | | Not performed |
| 28 | Korpelainen et al 2017[36] | Kuopio University Hospital, Finland | Single Centre | Adult  AML or HSCT FN | Temperature ≥38.3°C within 24 hours ANC <500/uL | 87 | Pathological | sCD14 | Univariate | Complications, microbiological | Septic shock, positive blood culture | AUC | 0.959 | | | | | | | NA | | | Not performed |
| 29 | Michel et al 2017[37] | University Medical Center of Mainz, Germany | Single Centre | BMT with high-dose chemotherapy | Temperature ≥38.3°C or two consecutive ≥38.0°C over 1 hour Leukocyte count < 500/uL | 44 | Pathological | PCT, CRP, sTREM-1, IL-8 | Univariate | Complications | Serious complications defined as cardiac failure, respiratory failure, renal failure, hypotension, altered mental state, ICU admission, arrhythmias, death | Sensitivity Specificity AUC | PCT: 0.80 0.76 0.9162 | | | | | | | NA | | | Not performed |
| 30 | Taj et al 2017[38] | Single center, Pakistan | Single Centre | Haematological malignancy FN | Temperature ≥38°C  ANC < 500/uL or ANC <1000/uL predicted decline to <500/uL | 226 | Clinical, pathological | MASCC score (symptoms, hypotension, COPD, previous fungal infection, dehydration, outpatient status, age) | Multivariate | Mortality, complications | Serious complications defined as cardiac failure, respiratory failure, renal failure, hypotension, altered mental state, ICU admission, arrhythmias, ECG changes, fungal infection, allergic reaction Mortality before neutropenic resolution | Sensitivity Specificity PPV | NA | | | | | | | 0.65 0.75 0.93 | | | Not performed |
| 31 | Wang et al 2017[39] | Singapore general hospital, Singapore | Single Centre | Age ≥ 16 years Solid tumour or lymphoma FN | Temperature ≥38.0°C ANC < 500/uL or ANC <1000/uL predicted decline to <500/uL | 120 | Clinical, pathological | MASCC score (symptoms, hypotension, COPD, previous fungal infection, dehydration, outpatient status, age), malaise subscale | Multivariate | Complications | Arrhythmia requiring treatment, severe bleeding requiring transfusion, confusion, heart failure, death, DIC, hypotension, ICU admission, persistence of blood culture positivity or breakthrough bacteraemia, IFI, allergic reaction, hospital readmission before fever resolution, renal failure requiring intervention, respiratory failure, sepsis or septic shock | Sensitivity Specificity PPV NPV Misclassification AUC | PROMASCC: 0.821 0.643 0.81 0.659 0.242 0.732 | | | | | | | MASCC: 0.936 0.381 0.737 0.762 0.258 0.658 | | | Not performed |
| 32 | Aagaard et al 2018[40] | Rigshospitalet, Copenhagen; Denmark | Single Centre | Solid cancer or DLBCL Standard first-line chemotherapy | Blood culture collected or death within 3 days of a neutrophil count less than 500/uL or leukocyte count less than 2000/uL In sensitivity analysis, Temperature ≥38.0 and neutrophils < 500/uL | 11229 | Clinical, pathological | Age, cancer type, cancer stage, chemotherapy type, albumin, eGFR, infection before chemotherapy, bilirubin, CRP | Multivariate | FN | Positive blood culture or death In sensitivity analysis, Temperature $\geq$38.0 and neutrophils < 500/uL | Harrell's C-statistic | 0.79 | | | | | | | NA | | | Not performed |
| 33 | Ahn et al 2018[41] | USA, UK, South Korea | Multi-centre | Age > 18 Malignancy FN Emergency department | Temperature ≥38°C  ANC < 1000/uL | 571 | Clinical, pathological | MASCC score (symptoms, hypotension, COPD, previous fungal infection, dehydration, outpatient status, age), CISNE score (ECOG, stress-induced hyperglycemia, COPD, CVS history, mucositis, monocyte count) | Multivariate | Mortality, complications | Serious complications (defined as respiratory failure, renal failure, hypotension, arrhythmias, DIC, death, ICU admission, other complications judged serious and clinically significant) Mortality | AUC Sensitivity Specificity PPV NPV PLR NLR | NA | | | | | | | MASCC:  0.772 0.9336 0.2689 0.8291 0.5161 1.28 0.25 | CISNE: 0.681 0.1239 0.9664 0.9333 0.2250 3.69 0.91 | | Not performed |
| 34 | Intke et al 2018[42] | Kuopio University Hospital, Finland | Single Centre | Adult Inpatients FN Intensive chemotherapy | Temperature ≥38.3°C or ≥38°C ≥1 hour ANC < 500/uL or ANC <1000/uL predicted decline to <500/uL | 86 | Pathological | IL1-Ra, CRP, PCT Day 0-2 | Univariate | Complications | Severe sepsis - sepsis with end organ dysfunction | Sensitivity Specificity PPV NPV ROC Younden's Index | D1 IL1-Ra Severe sepsis: 0.875 0.773 0.292 0.983 0.822 0.65 | | | | | | | NA | | | Not performed |
| 35 | Kauffmann-Guerrero et al 2018[43] | Single centre, Germany | Single Centre | SCLC  Treated at single institution | Temperature ≥38.3°C or ≥38°C ≥2 hours ANC < 500/uL | 52 | Pathological | CRP and neutrophils | Multivariate | FN | FN | Sensitivity Specificity PPV Wilks' lambda | 0.800 0.789 0.749 0.610 | | | | | | | NA | | | Not performed |
| 36 | Kelly et al 2018[44] | Brigham and Women’s Hospital, USA | Single Centre | FN | Temperature ≥38.3°C or ≥38°C ≥1 hour ANC <1000/uL | 39 | Pathological | MASCC 5 metabolic variables (not stated) RAD18, MAPKAPK3, JAG1 | Multivariate | Microbiological | Positive blood cultures | AUROC | Metabolic: 0.991  Genes: 0.961 | | | | | | | MASCC: 0.624 | | | Not performed |
| 37 | Li et al 2018[45] | Kaiser Permanente Southern California, USA | Single Centre | Adult  NHL, breast, lung, CRC, ovarian or gastric cancer | ICD-9 codes 780.6 and 288.0 in database within 7 days ANC <1000/uL and 780.6 Hospitalisation with neutropenia as primary diagnosis ICD-9 288.0 or ANC <1000/uL and bacterial/ fungal infection code | 15279 | Clinical, pathological | Age, GFR, WCC, cancer type, corticosteroid use, RDI ≥85%, chemotherapy use, obesity, diabetes, heart failure, COPD, rheumatoid disease, OA, other autoimmune disease, peptic ulcer disease, thyroid disorder, liver disease, HIV, recent dermatologic or mucosal condition | Multivariate | FN | FN 1st cycle | AUC | Novel model: 0.72 | | | | | | | Lyman model: 0.71 | | | Yes |
| 38 | Moon et al 2018[46] | National Cancer Center, South Korea | Single Centre | Age ≥ 18 years Chemotherapy within 30 days Stable patients only for CISNE evaluation FN Emergency department presentation | Temperature ≥38°C  ANC < 500/uL or ANC <1000/uL predicted decline to <500/uL | 400 | Clinical, pathological | MASCC score (symptoms, hypotension, COPD, previous fungal infection, dehydration, outpatient status, age), CISNE score (ECOG, stress-induced hyperglycemia, COPD, CVD history, mucositis, monocyte count) | Multivariate | Complications, microbiological | Serious complications defined as respiratory failure, renal failure, hypotension, altered mental state, ICU admission, acute heart dysfunction, procedures required to treat febrile illness, acute abdomen requiring urgent surgical or medical attention, IDC, major bleeding Bacteraemia | AUC C-index | NA | | | | | | | MASCC complication: 0.66 0.80  MASCC complication or bacteraemia C index 0.78  CISNE complication: AUC 0.64 | | | Not performed |
| 39 | Netterberg et al 2018[47] | Uppsala University Hospital, Sweden | Single Centre | Adult  Breast cancer Chemotherapy | ANC < 1000/uL Fever not defined | 49 | Pathological, clinical | CRP, IL-6 | Multivariate | FN | FN | NA | NA | | | | | | | NA | | | Not performed |
| 40 | Shimanuki et al 2018[48] | Kawasaki Municipal Kawasaki Hospital, Japan | Single Centre | Adult  TPF chemotherapy for head and neck cancer | Temperature ≥37.5°C ANC < 500/uL or ANC <1000/uL predicted decline to <500/uL | 50 | Pathological, clinical | AMC, ANC | Multivariate | FN | FN | Sensitivity Specificity PPV NPV PLR NLR OR AUC | 0.833 0.657 0.455 0.920 2.43 0.25 9.58 0.745 | | | | | | | NA | | | Not performed |
| 41 | García de Guadiana-Romualdo et al 2019[49] | University Hospital Santa Lucia, Spain | Single Centre | Adult Outpatients Chemotherapy for malignancy within 5 weeks prior to ED presentation | Temperature ≥38.3°C or ≥38°C ≥1 hour ANC < 500/uL or ANC <1000/uL predicted decline to <500/uL | 111 episodes, 102 patients | Clinical, pathological | MASCC score (symptoms, hypotension, COPD, previous fungal infection, dehydration, outpatient status, age), PCT, lipopolysaccharide binding protein | Multivariate, univariate | Complications, microbiological | Complications: Hypotension, respiratory failure, intensive care unit admission, DIC, confusion or altered mental status, CCF, bleeding requiring transfusion, arrythmia requiring treatment, renal failure, other clinically significant complication Bacteraemia | AUROC | Complications: PCT - 0.85, MASCC + LBP - 0.85  Bacteraemia: PCT: 0.86 | | | | | | | MASCC: Complications - 0.83 Bacteraemia - 0.74 | | | Not performed |
| 42 | Kim et al 2019[50] | Single centre, South Korea | Single Centre | Adult  Septic shock ED presentation Chemotherapy-Induced FN Treated with G-CSF | Temperature ≥38.3°C or 2 consecutive readings of ≥38°C in 2 hours ANC < 500/uL or expected to fall below 500/uL in 72 hours | 158 | Pathological, clinical | APACHE II score and PLR - both as individual models | Univariate/ Multivariate | Mortality | 1 month survival | AUROC Sensitivity Specificity PPV NPV | PLR: 0.666 0.894 0.462 0.829 0.600 | | | | | | | APACHE II: 0.730 0.868 0.636 0.861 0.651 | | | Yes |
| 43 | Kostic et al 2019[51] | University of Padua Hospital, Italy | Single Centre | Admission for high dose chemotherapy or ASCT FN Adult | Temperature ≥38.3°C or  Temperature ≥38°C for 1 hour ANC < 500/uL or <1000/ul and expected to decrease to < 500/uL | 28 | Pathological | Presepsin, PCT, IL-8, CRP | Univariate | Microbiological | Bacteraemia | AUROC Sensitivity Specificity PPV NPV Accuracy | PCT: 0.90 0.727 1.00 1.00 0.893 0.916 | | | | | | | NA | | | Not performed |
| 44 | Luo et al 2019[52] | Fujian Medical University Union Hospital, China | Single Centre | Haematological disease FN Age ≥14 Simultaneous collection of BCs and PCT | Temperature ≥38.3°C or ≥38°C ≥1 hour ANC < 500/uL or ANC <1000/uL predicted decline to <500/uL | 1466 | Pathological | PCT | Univariate | Microbiological | Bacteraemia Gram-negative bacteraemia Multidrug resistant gram-negative bacteremia | AUROC Sensitivity Specificity | Positive BC: 0.680 0.50 0.782 | Gram-negative BSI 0.703 0.552 0.768 | | | | | MDR GN BSI 0.601 0.726 0.511 | NA | | | Not performed |
| 45 | Perazzoli et al 2019[53] | Single centre, Brazil | Single Centre | FN Inpatient Abdominal/ anorectal infection site Haematological malignancy | Not stated | 74 episodes, 69 patients | Clinical, pathological | Baseline haematologic diagnosis, neutropenia severity, duration of neutropenia, therapeutic modality, diagnosis of abdominal or anorectal disease | Multivariate | Mortality | Inpatient mortality | AUROC | 0.82 | | | | | | | NA | | | Yes |
| 46 | Shilpakar et al 2019[54] | Civil Service Hospital, Nepal | Single Centre | Adult AML or ALL Chemotherapy FN | Temperature ≥38.3°C or ≥38°C for 1 hour ANC < 500/uL or ANC <1000/uL with prediction to be less than <500/uL | Not stated | Pathological | PCT, CRP | Univariate | Microbiological | Bacteraemia | AUROC Sensitivity Specificity | PCT:  0.90 0.91 0.69 | | | CRP: 0.59 Not reported Not reported | | | | NA | | | Not performed |
| 47 | Verlinden et al 2019[55] | Antwerp University Hospital | Single Centre | Acute leukaemia or stem cell transplant Chemotherapy | Standard Temperature ≥38.3°C or ≥38°C ≥2 hours ANC < 500/uL | 121 | Pathological | D0 and 2 CRP and PCT | Univariate | Microbiological | MDI, CDI, IFD, FUO | Sensitivity Specificity PPV NPV Efficiency | For IFI: 0.50 0.97 0.667 0.942 0.92 | | | | | | | NA | | | Not performed |
| 48 | Yang et al 2019[56] | Seoul St. May's Hospital, South Korea | Single Centre | Haematological malignancy Febrile episode PCT, CRP and serial blood cultures collected | Temperature ≥37.5°C ANC < 500/uL | 273 episodes | Pathological | PCT, CRP | Univariate | Microbiological | Bacteraemia | AUROC | PCT: 0.642 | | | | | | | NA | | | Not performed |
| 49 | Aagaard et al 2020[57] | Rigshospitalet, Copenhagen; Denmark | Single Centre | Solid cancers treated with chemotherapy Adult | Blood culture collected or death within 3 days of a neutrophil count less than 500/uL or leukocyte count less than 2000/uL In sensitivity analysis, T ≥38.0°C and neutrophils < 500/uL | 8076 | Clinical, pathological | FENCE score, chemotherapy type, concurrent radiotherapy, cycle number, previous FN or neutropenia, G-CSF use | Multivariate | FN | Blood culture collected or death within 3 days of a neutrophil count less than 500/uL or leukocyte count less than 2000/uL | Harrell's C-statistic | 0.75 | | | | | | | NA | | | Not performed |
| 50 | Chantharakhit et al 2020[58] | Buddhasothorn Hospital, Thailand | Single Centre | Stage I-III breast cancer | Temperature ≥38.5°C ANC < 500/uL or <1000/uL and expected to decrease to < 500/uL | 339 | Pathological, clinical | NLR | Multivariate | FN | FN | AUROC Sensitivity Specificity PPV NPV PLR NLR | 0.7626 0.6670 0.6450 0.11 0.967 1.88 0.52 | | | | | | | NA | | | Not performed |
| 51 | Cho et al 2020[59] | Hallym University Sacred Heart Hospital, South Korea | Single Centre | Age ≥18 Breast cancer Received chemotherapy | Temperature ≥38.3°C or ≥38°C ≥1 hour ANC < 500/uL or ANC <1000/uL predicted decline to <500/uL | 933 | Clinical, pathological | Eosinophil count, CEA, body surface area, lymphocyte count, neutrophil count, WCC, platelets | ML | FN | FN | AUROC Sensitivity Specificity Accuracy PPV NPV | 0.908 0.829 0.804 0.816 0.791 0.841 | | | | | | | NA | | | Not performed |
| 52 | Du et al 2020[60] | HCUP’s National Inpatient Sample and Nationwide Inpatient Sample, USA | Database | Adult Malignancy | ICD-9 codes 780.6 and 288.0 in database | 126013 | Clinical | Intubation and mechanical ventilation, respiratory failure, cardiac arrest and ventricular fibrillation, other aftercare, shock Top 5 ridge: other aftercare, respiratory failure, age, respiratory intubation and mechanical ventilation, acute and unspecified renal failure | ML | Mortality | Inpatient mortality | AUROC Sensitivity Specificity Precision Recall F1-score | 0.92 0.81 0.89 0.84 0.70 0.75 | | | | | | | NA | | | Not performed |
| 53 | Gulleen et al 2020[61] | University of Virgina Medical Center, USA | Single Centre | Adult Neutropenia | Temperature ≥38 ANC<500/uL | 1531 | Clinical, pathological | Temperature, heart rate, magnesium, platelets, haemoglobin | Multivariate | FN | FN | AUROC | 0.74 | | | | | | | 0.7 | | | Not performed |
| 54 | Halder et al 2020[62] | All India Institute of Medical Sciences, India | Single Centre | Age 1-60 years Haematological malignancy ANC < 500/uL FN | Temperature ≥38.3°C or ≥38°C ≥1 hour ANC < 500/uL | 52 episodes, 50 patients | Pathological | CRP and PCT at 0hr, 24hr, 48hr, 7-day, 14-day - all assessed as single variable models | Univariate | Microbiological | IFI, microbiologically documented infection | AUROC Sensitivity Specificity PPV NPV | PCT for IFI vs MDI-B: 0.766 0.625 0.875 Not stated 0.70 | | | CRP for IFI vs other fever: 0.685 1 0.48 0.333 1 | | | | NA | | | Not performed |
| 55 | Lappalainen et al 2020[63] | Kuopio University Hospital, Finland | Single Centre | Age ≥18 AML receiving chemotherapy FN | Temperature ≥38.3°C or ≥38°C ≥1 hour ANC < 500/uL or ANC predicted decline to <500/uL within 48 hours | 125 patients, 396 treatment episodes | Clinical | qSOFA ≥2 (BP, RR, confusion) | Multivariate | Mortality, complications | Infectious mortality ICU treatment | Sensitivity Specificity | NA | | | | | | | ICU admission: 0.700 0.979  Mortality: 1.000 0.971 | | | Not performed |
| 56 | Marín et al 2020[64] | Solon Espinosa Ayala, Ecuador | Single Centre | Critically ill cancer patients ICU admission FN Suspected infection | Not stated | 117 | Pathological | PCT | Univariate | Microbiological | Positive blood culture Positive gram-negative blood culture | AUROC Sensitivity Specificity PPV NPV | BSI: 0.76 0.714 0.682 0.588 0.789 | | | Gram-negative BSI: 0.80 0.581 0.919 0.72 0.859 | | | | NA | | | Not performed |
| 57 | Mohindra et al 2020[65] | Single center, India | Single Centre | Age ≥ 12 years Haematological or solid malignancy Chemotherapy-related FN | Temperature ≥38°C ≥ 1 hour ANC <1000/uL | 129 | Clinical, pathological | MASCC score (symptoms, hypotension, COPD, previous fungal infection, dehydration, outpatient status, age), CISNE score (ECOG, stress-induced hyperglycemia, COPD, CVS history, mucositis, monocyte count) | Multivariate | Mortality | 30-day mortality | AUC Sensitivity Specificity PPV NPV PLR NLR | NA | | | | | | | CISNE: 0.687 0.569 0.790 0.844 0.478  2.72 0.54 | MASCC: 0.675 0.581 0.651 0.769 0.437 1.66 0.64 | | Not performed |
| 58 | Odemis et al 2020[66] | Saglik Bilimleri University Hospital, Turkey | Single Centre | Age ≥ 18 years Infectious diseases, clinical microbiology or haematology service Case-control - FN or severe neutropenia without fever | ANC < 500/uL Temperature ≥ 38 | 88 | Pathological | CRP, PCT, lactate, MCP-1 | Univariate | Microbiological | BSI | AUROC | CRP: 0.834 | | | | | | | NA | | | Not performed |
| 59 | Peyrony et al 2020[67] | Santi-Louis Hospital, France | Single Centre | FN Age ≥ 18 years | Temperature ≥38.3°C or ≥38°C ≥1 hour ANC < 500/uL or ANC <1000/uL predicted decline to <500/uL | 249 | Clinical, pathological | MASCC score (symptoms, hypotension, COPD, previous fungal infection, dehydration, outpatient status, age) | Multivariate | Mortality, Complications | Inpatient mortality Serious complications defined as hypotension, respiratory failure, ICU admission, disseminated intravascular coagulation, confusion or altered mental state, CCF, bleeding requiring transfusion, arrhythmia requiring treatment, renal failure | AUC Sensitivity Specificity PPV NPV | NA | | | | | | | 0.67 0.78 0.43 0.676 0.566 | | | Not performed |
| 60 | Shimony et al 2020[68] | Rabin Medical Center, Israel | Single Centre | New acute leukaemia Age ≥ 18 during first induction | Temperature ≥38.3°C or ≥38°C for 1 hour ANC < 500/uL or ANC <1000/uL with prediction to be less than <500/uL | 138 | Pathological | CRP | Univariate | FN | FN | AUC Sensitivity Specificity | 0.68 0.92 0.30 | | | | | | | NA | | | Not performed |
| 61 | Alshari et al 2021[69] | King Abdullah University Hospital, Jordan | Single Centre | FN Receiving chemotherapy Treated with G-CSF | Not stated | 80 | Pathological | AMC | Univariate | Mortality, other | ANC difference from baseline D1-D6 Mortality or recurrence of FN | AUROC Sensitivity Specificity | * | | | | | | | NA | | | Not performed |
| 62 | Bhardwaj et al 2021[70] | Baystate Medical Center, USA | Single Centre | Age ≥18 Inpatient Cancer FN | Temperature ≥38.3°C within 24 hours ANC <500/uL | 193 | Clinical | MASCC score (symptoms, hypotension, COPD, previous fungal infection, dehydration, outpatient status, age) | Multivariate | Complications, mortality, microbiological | Inpatient death, ICU, goals of care discussion, respiratory failure, delirium, AKI requiring dialysis, septic shock requiring vasopressors, positive blood cultures | AUC | NA | | | | | | | Septic shock 0.764 Mortality 0.721 | | | Not performed |
| 63 | Cetintepe et al 2021[71] | University of Health Sciences Izmir Bozyaka Education and Research Hospital, Turkey | Single Centre | FN ICU admission Haematology | Temperature ≥38.3°C or ≥38°C ≥1 hour ANC < 500/uL or ANC <1000/uL predicted decline to <500/uL | 60 | Clinical, pathological | MASCC score (symptoms, hypotension, COPD, previous fungal infection, dehydration, outpatient status, age) SOFA score (respiratory, coagulation, bilirubin, blood pressure, pressor use, GCS, creatinine) qSOFA (respiratory rate, confusion, blood pressure) | Multivariate | Mortality | Mortality | Sensitivity Specificity | * | | | | | | | MASCC: 0.188 0.75 | | | Not performed |
| 64 | Chaftari et al 2021[72] | MD Anderson Cancer Center, USA | Single Centre | Cancer  ED presentation FN Serum lactate and PCT measured at presentation | Temperature ≥38°C or subjectively reported fever ANC < 500/uL | 550 | Clinical, pathological | MASCC, lactate, PCT | Multivariate, univariate | Mortality, microbiological, other | 14-day mortality, 30-day mortality, BSI, LOS ≥ 7 days | AUROC Specificity Sensitivity NPV PPV | 0.76 0.78 0.59 0.34 0.91 | | | | | | | MASCC 0.65 | | | Not performed |
| 65 | Garcia-Vidal et al 2021[73] | Hospital Clinic Barcelona, Spain | Single Centre | Haematological malignancy FN | Temperature ≥38°C ANC<500/uL | 3235 episodes, 349 patients | Clinical, pathological, microbiological | ML model without description | ML | Microbiological | GNB-MDR positive culture within 24 hours of FN onset | AUROC F1 Score Sensitivity Specificity NPV PPV | 0.7945 0.9670 0.4895 0.9886 0.9464 0.8246 | | | | | | | NA | | | Not performed |
| 66 | Monuszko et al 2021[74] | Single Centre, United States of America | Single Centre | Gynecological malignancy | Temperature ≥38.3°C or ≥38.0°C ≥1 hour ANC <1000/uL | 50 | Clinical, pathological | MASCC score (symptoms, hypotension, COPD, previous fungal infection, dehydration, outpatient status, age), CISNE score (ECOG, stress-induced hyperglycemia, COPD, CVD history, mucositis, monocyte count) | Multivariate | Mortality, Complications | Serious complications defined as hypotension, respiratory failure, confusion or altered mental state, CCF, bleeding requiring transfusion, arrhythmia requiring treatment, renal failure, inpatient death | Sensitivity Specificity PPV NPV | NA | | | | | | | MASCC + CISNE: 0.80 0.733 0.875 0.611 | | | Not performed |
| 67 | Moustafa et al 2021[75] | AL-Mowasat University Hospital, Syria | Single Centre | ≥14 years old FN Acute leukaemia | Temperature ≥38.3°C or ≥38°C ≥1 hour ANC < 500/uL or ANC <1000/uL predicted decline to <500/uL | 60 | Pathological | PSPN, PCT, CRP at day 1 and day 3 - six models assessed | Univariate | Microbiological | Confirmed infection on blood culture or local infection source | AUROC Specificity Sensitivity NPV PPV | 0.931 0.5 0.975 0.909 0.796 | | | | | | | NA | | | Not performed |
| 68 | Reyes Mondragón et al 2021[76] | Dr. Jose E. Gonzalez University Hospital of the Universidad Autonoma de Nuevo Leon, Mexico | Single Centre | Haematological malignancy FN Less than 24 hour arrival to ED | Temperature ≥38.3°C or ≥38°C ≥1 hour ANC < 500/uL | 81 episodes, 72 patients | Pathological | PCT, compared to MASCC | Univariate | Mortality, complications | Death as inpatient Sepsis (qSOFA >2) Septic shock | AUROC | PCT: Death - 0.810 Septic shock - 0.742 | | | | | | | MASCC: Death - 0.676 Septic shock - 0.713 | | | Not performed |
| 69 | Sereeaphinan et al 2021[77] | Songklanagarind Hospital, Thailand | Single Centre | Adult Chemotherapy FN | Temperature ≥38.3°C or ≥38°C for 1 hour ANC < 500/uL or ANC <1000/uL with prediction to be less than <500/uL | Not stated | Clinical, pathological | Hb, septic shock, AKI, mechanical ventilation | Multivariate | Mortality | 30-day mortality | AUROC | 0.8939 | | | | | | | NA | | | Yes |
| 70 | Yadav et al 2021[78] | All India Institute of Medical Sciences, India | Single Centre | >12 years old Haematologic or solid malignancy Chemotherapy-induced FN | ANC<1000/uL Temperature≥38 | 100 | Clinical, pathological | MASCC score (symptoms, hypotension, COPD, previous fungal infection, dehydration, outpatient status, age), PCT | Multivariate | Mortality | 30 day mortality | AUROC | PCT 0.664 | | | | | | | MASCC 0.586 | | | Not performed |
| 71 | Bozcuk et al 2022[79] | Multiple centres; Turkey | Multi-centre | Adult Solid cancer on chemotherapy | Temperature ≥38.3°C or ≥38°C ≥1 hour ANC < 500/uL or ANC <1000/uL predicted decline to <500/uL | 1909 patients, 4728 chemotherapy cycles | Pathological, clinical | ECOG, type of cancer, history of palliative radiotherapy, previous FN, chemotherapy cycle number, regimen risk, place of chemotherapy administration, pre-cycle lymphocyte count | Multivariate/ ML | FN | Temperature 38.3 or 38 ≥ 1 hour ANC < 500/uL or ANC <1000/uL predicted decline to <500/uL | AUROC Sensitivity Specificity | 0.87 NA NA | | | | | | | 0.78 0.91 0.40 | | | Not performed |
| 72 | Buchan et al 2022[80] | The Ottawa Hospital, Canada | Single Centre | Adult age ≥ 18 AlloHCT or ASCT | Temperature ≥38.3°C or ≥38°C ≥1 hour ANC < 500/uL | 81 | Clinical, pathological | HRV, TNF-alpha, IL-6, IL-7 | Multivariate | FN, complications | FN Infections positive on microbiology or radiographically (independent of whether FN developed or not) | AUC Sensitivity Specificity PPV NPV | 0.87 0.86 0.79 0.85 0.84 | | | | | | | NA | | | Not performed |
| 73 | Carcò et al 2022[81] | Mediterranean Institute of Oncology, Italy | Single Centre | Age ≥18 Myeloma or lymphoma ASCT | Temperature ≥38.3°C or ≥38°C for 1 hour ANC < 500/uL or ANC <1000/uL with prediction to be less than <500/uL | 49 | Pathological | WCC, CRP | Univariate | FN | FN | AUC | WCC 0.92 CRP 0.60 | | | | | | | NA | | | Not performed |
| 74 | Choi et al 2022[82] | Severeance Hospital, South Korea | Single Centre | Age ≥18  Malignancy FN | Temperature ≥38°C ANC < 500/uL or ANC <1000/uL predicted to decrease to < 500/uL | 378 | Clinical, pathological | qSOFA (mental state, blood pressure, RR), MPV | Multivariate | Mortality, complications | 28 day mortality Serious complications defined as hypotension, respiratory failure, ICU admission, disseminated intravascular coagulation, confusion or altered mental state, CCF, bleeding requiring transfusion, arrhythmia requiring treatment, renal failure | AUC | 0.729 | | | | | | | MASCC: 0.814 | | | Yes |
| 75 | Coyne et al 2022[83] | Two emergency departments in California, USA | Multi-centre | Adult FN | Temperature≥38°C ANC<1000/uL | 198 | Pathological | Procalcitonin >0.25 | Multivariate | Mortality, complications | Mortality ICU admission | Odds ratio Sensitivity Specificity NPV PPV | Mortality: 8.75 0.929 0.402 0.987 0.106 | | ICU: OR NA 1.00 0.399 1.00 0.081 | | | | | NA | | | Not performed |
| 76 | Intke et al 2022[84] | Kuopio University Hospital, Finland | Single Centre | Adult  FN AML post chemotherapy or ASCT | Temperature ≥38.3°C within 24 hours ANC <500/uL | 86 | Pathological | CK-18 | Univariate | Complications | Severe sepsis, septic shock | AUC | 0.767 | | | | | | | NA | | | Not performed |
| 77 | Kubo et al 2022[85] | Single centre, Japan | Single Centre | 18-74 years old ECOG 0-2 de novo AML | Temperature ≥38°C  ANC <500/uL | 51 | Pathological | D11 c-D-index | Multivariate | FN | ANC <500/uL Temperature ≥ 38 | Sensitivity Specificity | 0.684 0.685 | | | | | | | NA | | | Not performed |
| 78 | Ono et al 2022[86] | Two hospitals, Japan | Single Centre | Lung cancer FN | Temperature ≥38.3°C or ≥37.8°C ≥1 hour ANC <1000/uL | 49 | Clinical, pathological | MASCC and CISNE scores | Multivariate | Other | Failure to resume chemotherapy treatment | Sensitivity Specificity Younden Index | NA | | | | | | | MASCC ≤ 9 0.61 0.97 0.58 | | | Not performed |
| 79 | Padmanabhan et al 2022[87] | National center for cancer care and research; Qatar | Database | Age ≥ 18 years FN | Temperature ≥38.3°C ANC <500/uL | 1166 | Clinical, pathological | Sepsis: Age, region, BSI, Treatment phase, Diagnosis, Sex, Line infection, polymicrobial BSI, UTI MDRO: Age, Type BSI, line, diagnosis, treatment phase, region, sex, colitis, skin infection, UTI Death: Age, sepsis, BSI, Diagnosis | ML | Multiple Mortality, microbiological | Sepsis, MDRO, mortality | Accuracy Recall AUROC | Sepsis 0.67 0.95 0.84 | MDRO 0.83 0.96 0.91 | | | | | Mortality 0.76 0.86 0.88 | NA | | | Not performed |
| 80 | Shan et al 2022[88] | First Affiliated Hospital of Soochow University; China | Single Centre | Adult allo-HSCT Febrile Weekly PCT Complete clinical data and follow up information | Temperature ≥38.3°C or ≥38°C ≥1 hour Neutropenia not defined | 219 | Pathological, clinical | Diagnosis, disease status, months from diagnosis, MTC, PCT | Multivariate | Microbiological, Mortality | Documented infection BSI 100-day survival | AUROC C-index | CDI: 0.637 NA | BSI: 0.811 NA | | | | | Mortality 0.697 0.691 | NA | | | Yes |
| 81 | Venäläinen et al 2022[89] | Turku University Hospital and Tampere University Hospital; Finland | Single Centre | Adult  Non-haematological cancer | ANC<1000/uL Temperature≥38°C | 10473 | Clinical, pathological | Breast cancer, sarcoma, neutrophil count, thrombocyte count, use of taxanes, use of taxanes and monoclonal antibodies, use of topoisomerase inhibitors, use of antimetabolites, use of GCSF, relative dose intensity | ML | Microbiological | Neutropenic infection: ANC < 500/uL and CRP >10 within 5 days of this FN as defined | AUROC | 0.77 | | | | | | | New model:  0.75  Lyman: 0.53  Li: 0.70 | | | Not performed |
| 82 | Zatarah et al 2022a[90] | King Hussein Cancer Center, Jordan | Single Centre | Adult  Solid tumour or DLBCL Treatment-naïve | ANC <500/uL Temperature ≥38.2°C | 860 patients, 2870 chemotherapy cycles | Clinical, pathological | FENCE score, chemotherapy type, concurrent radiotherapy, cycle number, previous FN or neutropenia, G-CSF use | Multivariate | FN | ANC <500/uL Temperature ≥38.2 | AUROC | NA | | | | | | | 0.72 | | | Not performed |
| 83 | Zatarah et al 2022b[91] | King Hussein Cancer Center, Jordan | Single Centre | Adult  Solid tumour or DLBCL Treatment-naïve | ANC <500/uL Temperature ≥38.2°C | 918 | Clinical, pathological | Age, cancer type, cancer stage, chemotherapy type, albumin, eGFR, infection before chemotherapy, bilirubin, CRP | Multivariate | FN | ANC <500/uL Temperature ≥ 38.2 | AUROC | NA | | | | | | | 0.65 | | | Not performed |
| 84 | Zhu et al 2022[92] | PLA General Hospital; China | Single Centre | Adult  First cycle of etoposide chemotherapy Solid tumour or NHL | Temperature ≥38.3°C or ≥38°C ≥1 hour ANC < 1000/uL | 1554 | Clinical, pathological | KPS, Metastatic sites ≥3, heart disease, recent surgery, alkylating agents, Bilirubin, lymphocyte count | Multivariate | FN | FN or severe neutropenia | AUROC | 0.9 | | | | | | | NA | | | Yes |
| 85 | Barros et al 2023[93] | Complexo Hospitalar de Niteroi | Single Centre | Adult  ASCT | Temperature ≥38°C  ANC < 500/uL | 309 | Clinical | MASCC, EBMT, qSOFA and combination of these | Multivariate | Complication, microbiological, mortality | BSI, admission to ICU, mortality, complicated FN (BSI, ICU or death) | Sensitivity Specificity NPV PPV | No clear individual model better than others | | | | | | | MASCC 0.44 0.69 0.36 0.76 | | | Not performed |
| 86 | Erdem et al 2023[94] | 41 referral centres from 16 countries | Multicentre | ≥16 years FN Bacteraemia Initial BSI after hospital admission | Temperature ≥38.3°C or ≥38°C for 1 hour ANC < 500/uL or ANC <1000/uL with prediction to be less than <500/uL | 431 | Clinical, pathological | HR, qSOFA, appropriateness of antimicrobial treatment, UTI, Gram-positive BSI | Multivariate | Mortality | 30-day mortality | AUC Sensitivity Specificity | 0.821 0.812 0.65 | | | | | | | NA | | | Not performed |
| 87 | Frairia et al 2023[95] | University-Hospital Citta della Salute e della Scienza; Italy | Single Centre | Age ≥18 years AML FN | Temperature ≥38.3°C or ≥38°C ≥1 hour ANC < 500/uL or ANC <1000/uL with prevision at <500/uL | 630 FN | Clinical | qSOFA, National Early Warning Score | Univariate | Mortality Microbiological Complications | Time 0, 24, 48 hour: Mortality during FN Vasopressor requirement SIRS Respiratory failure Ventilation ICU admission | AUROC Odds ratio | No clear individual model better than others | | | | | | | NA | | | Not performed |
| 88 | Goto et al 2023[96] | Osaka Metropolitan University Graduate School of Medicine, Japan | Single Centre | FEC100, TC, eribulin mesylate, weekly paclitaxel | Temperature ≥37.5°C ANC <500/ul | 327 | Clinical, pathological | BMI, nutritional index, WCC, ANC, ALC, NLR | Univariate | FN | FN | AUC Sensitivity Specificity | BMI: 0.60 0.784 0.489 | | | | | | | NA | | | Not performed |
| 89 | Rattanathammethee et al 2023[97] | Chiang Mai University Hospital; Thailand | Single Centre | Aged ≥18  New diagnosis AML First induction 7+3 chemotherapy Adequate serial CBC during admission | Temperature ≥38.3°C or ≥38°C ≥1 hour ANC < 500/uL | 101 | Pathological | c-D-index, D-index, duration of grade 4 neutropenia, duration of profound neutropenia | Univariate | Microbiological | IFI - possible, probable, proven | Sensitivity Specificity PPV NPV ROC | D-index 0.813 0.835 0.482 0.959 0.937 | | | | c-D-index 0.688 0.682 0.289 0.921 0.802 | | | NA | | | Not performed |
| 90 | Thungthong et al 2023[98] | Rajavithi Hospital, Bangkok; Thailand | Single Centre | Adult  Lymphoma First-line chemotherapy | Blood culture collected or death within 3 days of a neutrophil count less than 500/uL or leukocyte count less than 2000/uL In sensitivity analysis, Temperature ≥38.0°C and neutrophils < 500/uL | 135 | Clinical, pathological | Age, cancer type, cancer stage, chemotherapy type, albumin, eGFR, infection before chemotherapy, bilirubin, CRP | Multivariate | FN | Blood culture collected or death within 3 days of a neutrophil count less than 500/uL or leukocyte count less than 2000/uL In sensitivity analysis, Temperature ≥38.0 and neutrophils < 500/uL | AUROC Sensitivity Specificity NPV NLR PLR Accuracy | NA | | | | | | | Lymphoma: 0.63 0.4643 0.7290 0.8387 0.73 1.71 0.6741 | DLBCL: 0.69 0.4737 0.7590 0.8630 0.69 1.97 0.7059 | | Not performed |
