# Supplementary material for: Clinical prediction models for febrile neutropenia and its outcomes: a systematic review
Source: Support Care Cancer. 2025 Jun 4;33(7):537. doi: 10.1007/s00520-025-09562-y (PMC12137469; doi:10.1007/s00520-025-09562-y)
Supplement: Supplementary file 2 — (DOCX 37.1 KB) [file 520_2025_9562_MOESM2_ESM.docx]

**Supplementary Table 2: PROBAST ROB and Applicability Assessment**

| **Paper Number** | **Reference** | **Risk of Bias** | | | | | **Applicability** | | | |
| --- | --- | --- | --- | --- | --- | --- | --- | --- | --- | --- |
|  |  | **Participants** | **Variables** | **Outcomes** | **Analysis** | **Overall** | **Participants** | **Variables** | **Outcomes** | **Overall** |
| 1 | Ahn et al 2013[9] | **+** | **+** | **-** | **-** | **-** | **+** | **+** | **+** | **+** |
| 2 | Gunderson et al 2013[10] | **-** | **-** | **-** | **-** | **-** | **-** | **-** | **-** | **-** |
| 3 | Kaya et al 2013[11] | **-** | **+** | **-** | **-** | **-** | **-** | **+** | **+** | **-** |
| 4 | Luz Fiuza et al 2013[12] | **-** | **-** | **+** | **-** | **-** | **-** | **+** | **+** | **-** |
| 5 | Lynn et al 2013[13] | **+** | **-** | **-** | **-** | **-** | **+** | **-** | **-** | **-** |
| 6 | Matsumoto et al 2013[14] | **+** | **○** | **-** | **-** | **-** | **+** | **+** | **-** | **-** |
| 7 | Meidani et al 2013[15] | **+** | **+** | **-** | **-** | **-** | **+** | **+** | **-** | **-** |
| 8 | Ribeiro et al 2013[16] | **+** | **+** | **-** | **-** | **-** | **+** | **+** | **-** | **-** |
| 9 | Schwenkglenks et al 2013[17] | **+** | **+** | **+** | **-** | **-** | **+** | **+** | **+** | **+** |
| 10 | Carmona-Bayonas et al 2014[18] | **+** | **+** | **-** | **-** | **-** | **+** | **+** | **+** | **+** |
| 11 | Chen et al 2014[19] | **-** | **+** | **-** | **-** | **-** | **-** | **+** | **-** | **-** |
| 12 | Gunalp et al 2014[20] | **+** | **-** | **-** | **-** | **-** | **+** | **+** | **-** | **-** |
| 13 | Patil et al 2014[21] | **-** | **+** | **-** | **-** | **-** | **-** | **+** | **-** | **-** |
| 14 | Pfeil et al 2014[22] | **+** | **+** | **+** | **-** | **-** | **-** | **+** | **+** | **-** |
| 15 | Shelburne et al 2014[23] | **+** | **+** | **+** | **-** | **-** | **+** | **+** | **+** | **+** |
| 16 | Bitar et al 2015[24] | **-** | **+** | **-** | **-** | **-** | **-** | **+** | **○** | **-** |
| 17 | Bozcuk et al 2015[25] | **+** | **+** | **+** | **-** | **-** | **+** | **+** | **+** | **+** |
| 18 | Carmona-Bayonas et al 2015[26] | **+** | **+** | **-** | **-** | **-** | **+** | **+** | **+** | **-** |
| 19 | Gardciade et al 2015[27] | **+** | **+** | **-** | **-** | **-** | **+** | **+** | **+** | **+** |
| 20 | Liu et al 2015[28] | **+** | **+** | **-** | **-** | **-** | **-** | **+** | **+** | **+** |
| 21 | Marini et al 2015[29] | **+** | **+** | **+** | **-** | **-** | **-** | **+** | **+** | **-** |
| 22 | Purhonen et al 2015[30] | **+** | **+** | **+** | **-** | **-** | **+** | **+** | **+** | **+** |
| 23 | Ahn et al 2016[31] | **+** | **+** | **-** | **-** | **-** | **+** | **+** | **+** | **-** |
| 24 | Fonseca et al 2016[32] | **+** | **+** | **-** | **+** | **-** | **+** | **+** | **+** | **-** |
| 25 | Coyne et al 2017[33] | **+** | **-** | **-** | **-** | **-** | **-** | **-** | **+** | **-** |
| 26 | Efe İris et al 2017[34] | **+** | **+** | **+** | **-** | **-** | **+** | **+** | **+** | **-** |
| 27 | Kim et al 2017[35] | **+** | **+** | **-** | **-** | **-** | **+** | **+** | **+** | **-** |
| 28 | Korpelainen et al 2017[36] | **+** | **+** | **+** | **-** | **-** | **+** | **+** | **+** | **+** |
| 29 | Michel et al 2017[37] | **+** | **+** | **-** | **-** | **-** | **+** | **+** | **+** | **-** |
| 30 | Taj et al 2017[38] | **+** | **+** | **-** | **-** | **-** | **-** | **+** | **+** | **-** |
| 31 | Wang et al 2017[39] | **-** | **+** | **-** | **-** | **-** | **-** | **+** | **+** | **-** |
| 32 | Aagaard et al 2018[40] | **+** | **+** | **-** | **-** | **-** | **+** | **+** | **-** | **-** |
| 33 | Ahn et al 2018[41] | **-** | **+** | **-** | **-** | **-** | **+** | **+** | **+** | **-** |
| 34 | Intke et al 2018[42] | **+** | **+** | **+** | **-** | **-** | **+** | **+** | **+** | **-** |
| 35 | Kauffmann-Guerrero et al 2018[43] | **-** | **+** | **+** | **-** | **-** | **-** | **+** | **+** | **-** |
| 36 | Kelly et al 2018[44] | **-** | **+** | **+** | **-** | **-** | **-** | **+** | **+** | **-** |
| 37 | Li et al 2018[45] | **-** | **+** | **-** | **-** | **-** | **-** | **+** | **-** | **-** |
| 38 | Moon et al 2018[46] | **-** | **+** | **-** | **-** | **-** | **-** | **+** | **+** | **-** |
| 39 | Netterberg et al 2018[47] | **-** | **+** | **-** | **-** | **-** | **-** | **+** | **-** | **-** |
| 40 | Shimanuki et al 2018[48] | **+** | **○** | **+** | **-** | **-** | **+** | **+** | **-** | **-** |
| 41 | García de Guadiana-Romualdo et al 2019[49] | **+** | **+** | **-** | **-** | **-** | **+** | **+** | **+** | **-** |
| 42 | Kim et al 2019[50] | **+** | **+** | **+** | **-** | **-** | **-** | **+** | **+** | **-** |
| 43 | Kostic et al 2019[51] | **+** | **+** | **+** | **-** | **-** | **+** | **+** | **+** | **+** |
| 44 | Luo et al 2019[52] | **-** | **+** | **+** | **-** | **-** | **-** | **+** | **+** | **-** |
| 45 | Perazzoli et al 2019[53] | **-** | **-** | **+** | **-** | **-** | **-** | **-** | **+** | **-** |
| 46 | Shilpakar et al 2019[54] | **+** | **+** | **+** | **-** | **-** | **+** | **+** | **+** | **+** |
| 47 | Verlinden et al 2019[55] | **+** | **+** | **+** | **-** | **-** | **+** | **+** | **+** | **+** |
| 48 | Yang et al 2019[56] | **-** | **+** | **+** | **-** | **-** | **-** | **+** | **+** | **-** |
| 49 | Aagaard et al 2020[57] | **+** | **+** | **-** | **-** | **-** | **+** | **-** | **-** | **-** |
| 50 | Chantharakhit et al 2020[58] | **-** | **+** | **+** | **-** | **-** | **-** | **+** | **+** | **-** |
| 51 | Cho et al 2020[59] | **+** | **+** | **+** | **-** | **-** | **+** | **+** | **+** | **+** |
| 52 | Du et al 2020[60] | **+** | **-** | **-** | **-** | **-** | **+** | **-** | **+** | **-** |
| 53 | Gulleen et al 2020[61] | **-** | **+** | **-** | **-** | **-** | **-** | **+** | **+** | **-** |
| 54 | Halder et al 2020[62] | **+** | **-** | **-** | **-** | **-** | **+** | **-** | **+** | **-** |
| 55 | Lappalainen et al 2020[63] | **+** | **+** | **+** | **-** | **-** | **+** | **+** | **+** | **+** |
| 56 | Marín et al 2020[64] | **-** | **+** | **+** | **-** | **-** | **-** | **+** | **+** | **-** |
| 57 | Mohindra et al 2020[65] | **+** | **+** | **+** | **-** | **-** | **-** | **+** | **+** | **+** |
| 58 | Odemis et al 2020[66] | **-** | **+** | **+** | **-** | **-** | **-** | **+** | **+** | **-** |
| 59 | Peyrony et al 2020[67] | **+** | **+** | **-** | **-** | **-** | **+** | **+** | **+** | **+** |
| 60 | Shimony et al 2020[68] | **+** | **+** | **+** | **-** | **-** | **+** | **+** | **+** | **+** |
| 61 | Alshari et al 2021[69] | **-** | **+** | **-** | **-** | **-** | **-** | **+** | **-** | **-** |
| 62 | Bhardwaj et al 2021[70] | **-** | **+** | **-** | **-** | **-** | **+** | **+** | **-** | **-** |
| 63 | Cetintepe et al 2021[71] | **+** | **+** | **+** | **-** | **-** | **-** | **+** | **+** | **-** |
| 64 | Chaftari et al 2021[72] | **-** | **-** | **+** | **-** | **-** | **-** | **-** | **+** | **-** |
| 65 | Garcia-Vidal et al 2021[73] | **+** | **+** | **+** | **-** | **-** | **+** | **+** | **+** | **+** |
| 66 | Monuszko et al 2021[74] | **+** | **+** | **-** | **-** | **-** | **+** | **+** | **+** | **-** |
| 67 | Moustafa et al 2021[75] | **-** | **+** | **-** | **-** | **-** | **-** | **-** | **-** | **-** |
| 68 | Reyes Mondragón et al 2021[76] | **+** | **+** | **+** | **-** | **-** | **+** | **+** | **+** | **+** |
| 69 | Sereeaphinan et al 2021[77] | **+** | **-** | **+** | **-** | **-** | **+** | **-** | **+** | **-** |
| 70 | Yadav et al 2021[78] | **+** | **+** | **+** | **-** | **-** | **-** | **+** | **+** | **+** |
| 71 | Bozcuk et al 2022[79] | **+** | **+** | **+** | **-** | **-** | **+** | **+** | **+** | **+** |
| 72 | Buchan et al 2022[80] | **-** | **+** | **-** | **-** | **-** | **-** | **+** | **-** | **-** |
| 73 | Carcò et al 2022[81] | **+** | **+** | **+** | **-** | **-** | **+** | **+** | **+** | **+** |
| 74 | Choi et al 2022[82] | **+** | **+** | **-** | **-** | **-** | **+** | **+** | **+** | **+** |
| 75 | Coyne et al 2022[83] | **+** | **+** | **+** | **-** | **-** | **+** | **+** | **+** | **○** |
| 76 | Intke et al 2022[84] | **+** | **+** | **+** | **-** | **-** | **+** | **+** | **+** | **+** |
| 77 | Kubo et al 2022[85] | **+** | **-** | **+** | **-** | **-** | **+** | **+** | **+** | **-** |
| 78 | Ono et al 2022[86] | **+** | **+** | **+** | **-** | **-** | **+** | **-** | **+** | **-** |
| 79 | Padmanabhan et al 2022[87] | **-** | **-** | **-** | **-** | **-** | **+** | **+** | **+** | **-** |
| 80 | Shan et al 2022[88] | **-** | **+** | **-** | **-** | **-** | **-** | **+** | **-** | **-** |
| 81 | Venäläinen et al 2022[89] | **+** | **+** | **-** | **-** | **-** | **+** | **-** | **-** | **-** |
| 82 | Zatarah et al 2022a[90] | **+** | **+** | **+** | **-** | **-** | **+** | **-** | **+** | **-** |
| 83 | Zatarah et al 2022b[91] | **+** | **+** | **+** | **-** | **-** | **+** | **+** | **+** | **+** |
| 84 | Zhu et al 2022[92] | **-** | **+** | **-** | **-** | **-** | **-** | **+** | **-** | **-** |
| 85 | Barros et al 2023[93] | **+** | **+** | **-** | **-** | **-** | **+** | **+** | **+** | **+** |
| 86 | Erdem et al 2023[94] | **+** | **-** | **+** | **-** | **-** | **-** | **-** | **+** | **-** |
| 87 | Frairia et al 2023[95] | **+** | **+** | **+** | **-** | **-** | **+** | **+** | **+** | **+** |
| 88 | Goto et al 2023[96] | **+** | **+** | **-** | **-** | **-** | **+** | **+** | **-** | **-** |
| 89 | Rattanathammethee et al 2023[97] | **+** | **+** | **+** | **-** | **-** | **+** | **+** | **+** | **+** |
| 90 | Thungthong et al 2023[98] | **+** | **+** | **-** | **-** | **-** | **+** | **+** | **-** | **-** |
