# Supplementary material for: Clinical prediction models for febrile neutropenia and its outcomes: a systematic review
Source: Support Care Cancer. 2025 Jun 4;33(7):537. doi: 10.1007/s00520-025-09562-y (PMC12137469; doi:10.1007/s00520-025-09562-y)
Supplement: Supplementary file 3 — (DOCX 16.3 KB) [file 520_2025_9562_MOESM3_ESM.docx]

**Pubmed**

("neutropen*"[Title/Abstract] OR "neutropaen*"[Title/Abstract] OR "granulocytopen*"[Title/Abstract] OR "granulocytopaen*"[Title/Abstract]) AND ("febrile"[Title/Abstract] OR "fever"[Title/Abstract]) AND ("clinical decision support system"[All Fields] OR "electronic health records"[MeSH Terms] OR "decision support systems, clinical"[MeSH Terms] OR "Medical Informatics Applications"[MeSH Terms] OR "risk score"[Title/Abstract] OR "scor*"[Title/Abstract] OR "predic*"[Title/Abstract] OR ("Artificial Intelligence"[MeSH Terms] OR "Machine learning"[MeSH Terms] OR ("deep learning"[MeSH Terms] OR ("deep"[All Fields] AND "learning"[All Fields]) OR "deep learning"[All Fields]) OR (("artificial"[All Fields] OR "artificially"[All Fields]) AND ("neural networks, computer"[MeSH Terms] OR ("neural"[All Fields] AND "networks"[All Fields] AND "computer"[All Fields]) OR "computer neural networks"[All Fields] OR ("neural"[All Fields] AND "network"[All Fields]) OR "neural network"[All Fields])) OR (("natural"[All Fields] OR "naturally"[All Fields] OR "naturals"[All Fields] OR "nature"[MeSH Terms] OR "nature"[All Fields] OR "nature s"[All Fields] OR "natures"[All Fields]) AND ("language"[MeSH Terms] OR "language"[All Fields] OR "languages"[All Fields] OR "language s"[All Fields] OR "programming languages"[MeSH Terms] OR ("programming"[All Fields] AND "languages"[All Fields]) OR "programming languages"[All Fields]) AND ("process"[All Fields] OR "processe"[All Fields] OR "processed"[All Fields] OR "processes"[All Fields] OR "processing"[All Fields] OR "processings"[All Fields])) OR (("thinking"[MeSH Terms] OR "thinking"[All Fields] OR "think"[All Fields] OR "thinks"[All Fields] OR "thinkings"[All Fields]) AND ("computer systems"[MeSH Terms] OR ("computer"[All Fields] AND "systems"[All Fields]) OR "computer systems"[All Fields] OR ("computer"[All Fields] AND "system"[All Fields]) OR "computer system"[All Fields])) OR (("fuzziness"[All Fields] OR "fuzzy"[All Fields]) AND ("expert systems"[MeSH Terms] OR ("expert"[All Fields] AND "systems"[All Fields]) OR "expert systems"[All Fields] OR ("expert"[All Fields] AND "system"[All Fields]) OR "expert system"[All Fields])) OR ("ieee trans evol comput"[Journal] OR "evol comput"[Journal] OR ("evolutionary"[All Fields] AND "computation"[All Fields]) OR "evolutionary computation"[All Fields]) OR (("chimera"[MeSH Terms] OR "chimera"[All Fields] OR "hybrid"[All Fields] OR "hybrids"[All Fields] OR "hybrid s"[All Fields] OR "hybridation"[All Fields] OR "hybridisations"[All Fields] OR "hybridise"[All Fields] OR "hybridised"[All Fields] OR "hybridises"[All Fields] OR "hybridising"[All Fields] OR "hybridity"[All Fields] OR "hybridization, genetic"[MeSH Terms] OR ("hybridization"[All Fields] AND "genetic"[All Fields]) OR "genetic hybridization"[All Fields] OR "hybridisation"[All Fields] OR "hybridizations"[All Fields] OR "hybridize"[All Fields] OR "hybridized"[All Fields] OR "hybridizes"[All Fields] OR "hybridizing"[All Fields] OR "nucleic acid hybridization"[MeSH Terms] OR ("nucleic"[All Fields] AND "acid"[All Fields] AND "hybridization"[All Fields]) OR "nucleic acid hybridization"[All Fields] OR "hybridization"[All Fields]) AND ("intelligence"[MeSH Terms] OR "intelligence"[All Fields] OR "intelligences"[All Fields] OR "intelligent"[All Fields] OR "intelligently"[All Fields] OR "intelligibilities"[All Fields] OR "intelligibility"[All Fields] OR "intelligible"[All Fields]) AND ("drug delivery systems"[MeSH Terms] OR ("drug"[All Fields] AND "delivery"[All Fields] AND "systems"[All Fields]) OR "drug delivery systems"[All Fields] OR "system"[All Fields] OR "system s"[All Fields] OR "systems"[All Fields])) OR ("Artificial Intelligence"[MeSH Terms] OR ("artificial"[All Fields] AND "intelligence"[All Fields]) OR "Artificial Intelligence"[All Fields]) OR ("Machine learning"[MeSH Terms] OR ("machine"[All Fields] AND "learning"[All Fields]) OR "Machine learning"[All Fields])))

**EMBASE**

('clinical decision support system' OR (electronic AND medical AND records) OR (electronic AND medical AND record) OR (medical AND record, AND electronic) OR (medical AND records, AND electronic) OR (electronic AND health AND record) OR (medical AND records, AND computerized) OR (medical AND record, AND computerized) OR (computerized AND medical AND record) OR (computerized AND medical AND records) OR (clinical AND decision AND support) OR (medical AND informati* AND applicatio*) OR 'risk score':ab,ti OR scor*:ab,ti OR predic*:ab,ti OR (intelligence, AND artificial) OR (computational AND intelligence) OR (intelligence, AND computational) OR (machine AND intelligence) OR (intelligence, AND machine) OR (computer AND reasoning) OR (reasoning, AND computer) OR (artificial AND intelligence) OR (computer AND vision AND systems) OR (computer AND vision AND system) OR (deep AND learning) OR (machine AND learning) OR 'natural language processing' OR (thinking AND computer AND system) OR (fuzzy AND expert AND system) OR (evolutionary AND computation) OR (hybrid AND intelligent AND system) OR 'artificial intelligence' OR 'machine learning') AND (neutropen*:ab,ti OR neutropaen*:ab,ti OR granulocytopen*:ab,ti OR granulocytopaen*:ab,ti) AND (fever:ab,ti OR febrile:ab,ti)

**SCOPUS**

(TITLE-ABS-KEY(fever OR febrile) AND TITLE-ABS-KEY(neutropenia OR neutropenic OR neutropaenia OR neutropaenic OR granulocytopenia OR granulocytopenic OR granulocytopaenia OR granulocytopaenic)) AND (("clinical decision support system" OR (electronic AND medical AND records) OR (electronic AND medical AND record) OR (medical AND record, AND electronic) OR (medical AND records, AND electronic) OR (electronic AND health AND record) OR (medical AND records, AND computerized) OR (medical AND record, AND computerized) OR (computerized AND medical AND record) OR (computerized AND medical AND records) OR (clinical AND decision AND support) OR (medical AND information AND application) OR TITLE-ABS-KEY(risk score) OR TITLE-ABS-KEY(score OR scores) OR TITLE-ABS-KEY(predict OR prediction OR predicts) OR (intelligence, AND artificial) OR (computational AND intelligence) OR (intelligence, AND computational) OR (machine AND intelligence) OR (intelligence, AND machine) OR (computer AND reasoning) OR (reasoning, AND computer) OR (artificial AND intelligence) OR (computer AND vision AND systems) OR (computer AND vision AND system) OR (deep AND learning) OR (machine AND learning) OR "natural language processing" OR (thinking AND computer AND system) OR (fuzzy AND expert AND system) OR (evolutionary AND computation) OR (hybrid AND intelligent AND system) OR "artificial intelligence" OR "machine learning"))

**WEB OF SCIENCE**

(AB=(fever OR febrile) OR TI=(fever OR febrile)) AND (TI=(neutropenia OR neutropenic OR neutropaenia OR neutropaenic OR granulocytopenia OR granulocytopenic OR granulocytopaenia OR granulocytopaenic) OR (AB=(neutropenia OR neutropenic OR neutropaenia OR neutropaenic OR granulocytopenia OR granulocytopenic OR granulocytopaenia OR granulocytopaenic))) AND (ALL=(("clinical decision support system" OR (electronic medical recor*) OR (electronic health recor*) OR (compute* medical recor*) OR (clinical decision support) OR (medical information application) OR (artificial intelligence) OR (computational intelligence) OR (machine intelligence) OR (computer reasoning) OR (reasoning computer) OR (computer vision syste*) OR (deep learning) OR (machine learning) OR "natural language processing" OR (thinking computer system) OR (fuzzy expert system) OR (evolutionary computation) OR (hybrid intelligent system) OR "artificial intelligence" OR "machine learning")) OR TI=(risk score) OR AB=(risk score) OR TI=(scor*) OR AB=(scor*) OR TI=(predic*) OR AB=(predic*))
